# Optical Configuration Effect on the Structure and Reactivity of Diastereomers Revealed by Spin Effects and Molecular Dynamics Calculations

**DOI:** 10.3390/ijms23010038

**Published:** 2021-12-21

**Authors:** Aleksandra A. Ageeva, Alexander B. Doktorov, Olga Yu. Selyutina, Ilya M. Magin, Margarita G. Ilyina, Sophia S. Borisevich, Ruslan Yu. Rubtsov, Sergey L. Khursan, Alexander A. Stepanov, Sergey F. Vasilevsky, Nikolay E. Polyakov, Tatyana V. Leshina

**Affiliations:** 1 Voevodsky Institute of Chemical Kinetics and Combustion, 630090 Novosibirsk, Russia; al.ageeva@gmail.com (A.A.A.); olga.gluschenko@gmail.com (O.Y.S.); magin@kinetics.nsc.ru (I.M.M.); stepanov@kinetics.nsc.ru (A.A.S.); vasilev@kinetics.nsc.ru (S.F.V.); polyakov@kinetics.nsc.ru (N.E.P.); leshina@ngs.ru (T.V.L.); 2 Department of Natural Sciences, Department of Physics, Novosibirsk State University, 630090 Novosibirsk, Russia; 3 Ufa Institute of Chemistry, Ufa Federal Research Centre of the Russian Academy of Sciences, 450054 Ufa, Russia; margarita.kondrova@yandex.ru (M.G.I.); monrel@mail.ru (S.S.B.); khursansl@gmail.com (S.L.K.); 4 The Institute of Problems of Chemical Physics of the Russian Academy of Sciences, 142432 Chernogolovka, Russia; mc9ybkpv@gmail.com

**Keywords:** chiral linked systems, diastereomers, hyperpolarization, electron transfer, spin selectivity, molecular dynamics, magnetic dipole–dipole interaction of electrons

## Abstract

The peculiarities of spin effects in photoinduced electron transfer (ET) in diastereomers of donor-acceptor dyads are considered in order to study the influence of chirality on reactivity. Thus, the spin selectivity—the difference between the enhancement coefficients of chemically induced dynamic nuclear polarization (CIDNP)—of the dyad’s diastereomers reflects the difference in the spin density distribution in its paramagnetic precursors that appears upon UV irradiation. In addition, the CIDNP coefficient itself has demonstrated a high sensitivity to the change of chiral centers: when one center is changed, the hyperpolarization of all polarized nuclei of the molecule is affected. The article analyzes the experimental values of spin selectivity based on CIDNP calculations and molecular dynamic modeling data in order to reveal the effect of optical configuration on the structure and reactivity of diastereomers. In this way, we succeeded in tracing the differences in dyads with L- and D-tryptophan as an electron donor. Since the replacement of L-amino acid with D-analog in specific proteins is believed to be the cause of Alzheimer’s and Parkinson’s diseases, spin effects and molecular dynamic simulation in model dyads can be a useful tool for investigating the nature of this phenomenon.

## 1. Introduction

**Figure 1:**
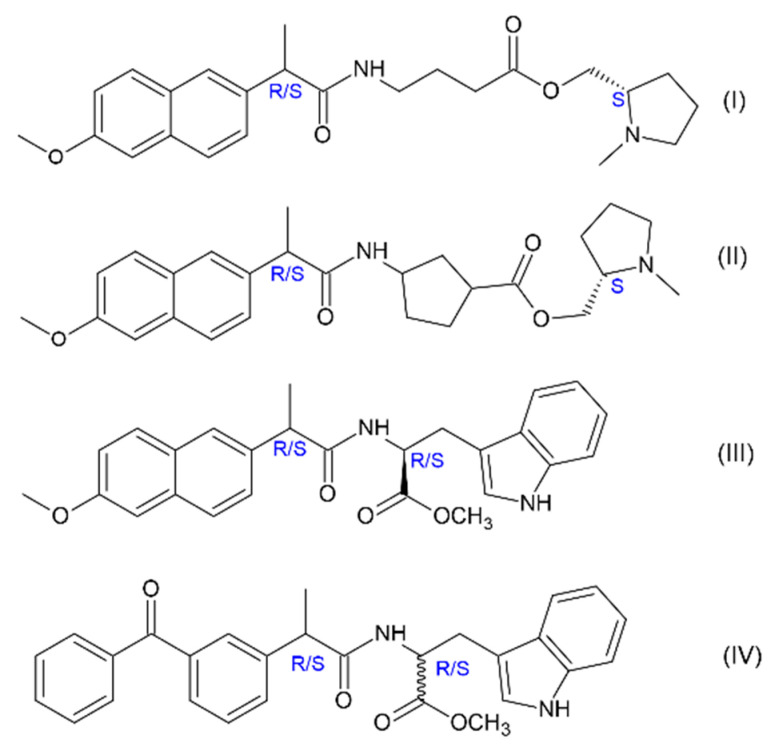
Chemical structure of chiral systems demonstrating spin selectivity.

Chirality plays a crucial role in many issues, beginning from the fundamental problem of the origin of life on the Earth up to the development of new functional materials [1]. Biomolecules such as proteins and DNA are known to consist of L-amino acids and D-sugars. Until recently, the homochirality of proteins was believed to be maintained during the lifespan of an organism. However, proteins with D-amino acids have been detected in various tissues, and their amounts have been shown to increase with organism aging. According to recent studies, the replacement of L-amino acids by D-analogs during aging is one of the causes of Alzheimer’s and Parkinson’s [1,2]. It was found that optical isomers may undergo so-called chiral inversion—conversion of one optical configuration to another one under various conditions, e.g., change of temperature, solvent, under UV irradiation, action of enzymes and other chiral substances, etc. [1]. On the other hand, the chirality of biomolecules also produces rigid requirements on the pharmaceutical industry, since the optical isomers of drugs (enantiomers) often have different and sometimes opposite therapeutic activities [3]. Currently, more than half the drugs on the market are chiral compounds, and most of them are still produced in the form of racemates (equimolar mixtures of two enantiomers) [3]. Numerous biochemical studies do not provide a final answer to the question of why enantiomers with identical physico-chemical properties demonstrate such differences in living organism [4]. Thus, the difference in biological activity of enantiomers of both chiral drugs (xenobiotics) and amino acids (structural components of biomolecules) are challenging problems. Certain successes in this direction in the last decade are associated with the use of linked systems with two chiral centers [5]. Investigations of model systems—dyads where molecule of chiral drug linked with chiral electron donor seem to be promising to address this problem since elementary processes in such donor–acceptor dyads are believed to simulate binding of drug molecule with amino acid residue in active site of enzyme. For example, spin chemistry and photochemistry studies of dyads involving naproxen enantiomers linked with (*S*)-N-methylpyrrolidine (I, II) and (*S*)-tryptophan (III) (Figure 1) revealed several peculiarities of electron transfer (ET)—stereo- and spin selectivity [6]. Recent studies have also shown spin selectivity in dyads where enantiomers of naproxen and ketoprofen linked with (*R*)-tryptophan (III and IV).

It is worth noting that representatives of non-steroidal anti-inflammatory drugs (NSAIDs), derivatives of propionic acid, especially naproxen, are among the most frequently prescribed medicines that exist in two enantiomeric forms with different therapeutic effects [3]. Stereo-selectivity of ET processes in dyads with (*R*)- and (*S*)-naproxen and another NSAID, ketoprofen, and its relevance to biochemical studies have been described in detail [6,7,8,9].

This report aims to discuss the other peculiarity of chiral systems—spin selectivity—and highlight the relationship between this phenomenon and optical configuration of the dyad. Spin selectivity is the difference in CIDNP coefficients of diastereomers [10]. The importance of this phenomenon is that the observed difference was explained by the difference in the distribution of spin density in paramagnetic precursors of diastereomers [10,11]. The latter also suggests a difference in the distribution of electron density in diastereomers. This is an important conclusion, in light of the abovementioned assumption, that processes occurring in diastereomers can simulate interaction of a chiral drug with chiral amino acid residues in the active sites of enzymes and receptors. If we consider the situation in this direction, then the difference in the distribution of electron density can be one of the real reasons for the differences in the therapeutic activity of drug enantiomers. It is well known that interactions between drug and amino acids at active sites involve charge redistribution.

Since the spin selectivity phenomenon belongs to the area of spin effects, we should give a brief overview of chemically induced dynamic nuclear polarization (CIDNP). The CIDNP is the manifestation in NMR spectra of the products of radical reactions as signals of nuclei with the spin state populations different from Boltzmann (hyperpolarization). The phases (emission or enhanced absorption) and intensities of these signals depend on certain parameters, such as hyperfine interaction (HFI) constants, the difference in g-factors of partners in a radical pair (RP) or biradical, the multiplicity of RP precursors, and a few more parameters [12]. In essence, CIDNP effects reflect the difference in the recombination probability of RPs with α_N_ and β_N_ nuclear spin projections on the magnetic field direction. The source of this difference is the difference in the energy of electron–nuclear interaction in RPs and in the Larmor precession frequencies of radical partners. The spin Hamiltonian describing these interactions in high magnetic field is presented below:

(1) $$\hat{H}=g_{1}\beta B{\hat{S}}_{1z}+g_{2}\beta B{\hat{S}}_{2z}+\underset{i}{\sum }a_{1i}{\hat{S}}_{1z}{\hat{I}}_{iz}+\underset{k}{\sum }a_{2k}{\hat{S}}_{2z}{\hat{I}}_{kz}$$

where *g*_1_ and *g*_2_ are g-factors of electrons, *β* is the Bohr magneton, *B* is external magnetic field induction, *S_1z_*, *S_2z_* and *I_iz_*, *I_kz_* are electron and nuclear spins operators (projection on *B* direction), and a is the HFI constant. As a result of the analysis of the CIDNP effects, information can be obtained on the radical stages of the process, in particular, on the spin density distribution in the radical precursors of the products. This information on the spin density distribution in the paramagnetic forms of drugs and amino acids can be practically significant for drug–receptor binding known to involve charge transfer processes. It should also be noted that the role of the external magnetic field in the processes of chiral enrichment has already been predicted [13].

Spin selectivity is referred to as difference in the CIDNP enhancement coefficients calculated for one pair of radicals. In the case of studied dyads, it is a biradical-zwitterion (BZ). The CIDNP enhancement coefficient for a specific proton in dyad is equal to the intensity of the polarized proton signal (*I_pol_*) divided by the intensity of its signal in the equilibrium NMR spectrum (*I_eq_*) and the concentration of biradical-zwitterion (BZ) formed as result of intramolecular ET [10]. For convenience, further, the ratio of CIDNP enhancement coefficients for diastereomers of dyads will be used:

(2) $$K_{RS/SS}=\frac{I_{pol}^{RS}I_{eq}^{SS}{\left[BZ\right]}_{SS}}{I_{eq}^{RS}I_{pol}^{SS}{\left[BZ\right]}_{RS}}$$

This value *K_RS/SS_* is the spin selectivity. The first systematic study of spin selectivity showed that the value of *K_RS/SS_* in dyads II and III is about 2. Authors have listed several reasons of this phenomenon and have arrived at the conclusion that the origin of spin selectivity is the difference of HFI constants in biradical-zwitterions of diastereomers [10,11]. More recent studies of a model system involving enantiomers of another NSAID—ketoprofen linked with enantiomers of tryptophan (set of (*S,S*)-, (*S,R*)- and (*R,S*)-optical configurations) IV—showed almost tenfold difference in CIDNP enhancement coefficients.

According to the literature data [5,12] the differences in the stereoselectivity of electron transfer in (*R,S*)- and (*S,S*)-diastereomers in similar systems vary within 25–40%. Taking into account that the CIDNP coefficient is associated with the manifestation of ET mechanism, it is difficult to imagine that the contributions of the ET mechanism can differ for (*S,S*)- and (*R,S*)-configurations by almost an order of magnitude. On the other hand, dyad IV is a system with two chromophores, and the prevailing mechanism of fluorescence quenching is resonance energy transfer (RET) [13,14]. According to [13], the stereoselectivity of singlet–singlet energy transfer in diastereomers hardly reaches 2, and our own data for dyad IV gives a value of 1.6 [14]. These differences in BZ concentrations in Equation (2), caused by RET stereoselectivity, could not provide an observable difference in the values of the CIDNP coefficients for the (*S,S*)- and (*R,S*)-configurations. Correspondingly, the difference in the CIDNP coefficients of the (*S,S*)-, (*R,S*)-, and (*S,R*)-configurations of dyad IV is associated with anomalously low hyperpolarization of the (*R,S*)- and (*S,R*)-enantiomers. This reasoning leads to the conclusion that the interpretation of spin selectivity associated with the difference in HFI constants in BZ of diastereomers is not the only one.

In order to trace some patterns in the abovementioned difference in the spin effects of optical isomers of dyads I–IV, a comprehensive analysis of CIDNP of all polarized protons and its spin selectivity will be carried out. This consideration involves the calculation of CIDNP in diastereomers of dyads in accordance with the S-T_0_ approximation using data from molecular dynamics and quantum chemical calculations.

In addition, an alternative explanation of the hyperpolarization will be given, involving magnetic dipole–dipole interaction of electrons in the spin Hamiltonian (1). An attempt to use the dipole–dipole electron–electron magnetic interaction to explain the difference in the CIDNP coefficients in the diastereomers of the dyad IV was already made in the work [14], but it was the CIDNP calculation without taking into account BZ lifetime. In this article, the CIDNP formed in the biradical-zwitterion within the framework of the two-position model will be calculated taking into account the electron dipole–dipole interaction when BZ is in and out of the reaction zone.

Thus, consideration the different efficiency of spin effects in diastereomers of dyads I–IV together with comparison of experimental results with molecular simulating data seems to be promising to discover the influence of optical configuration on the structure and the reactivity of donor–acceptor dyads. In particular, the analysis of these data for diastereomers of dyads III and IV with D- and L- residues of tryptophan, including the abnormally weak hyperpolarization in the (*R,S*)- and (*S,R*)-enantiomers of dyad IV, can shed light on the nature of the differences between systems with optical isomers of tryptophan. The addressing to model systems in this case is especially important, since the structures of certain proteins and peptides found in living systems become highly disordered when L-amino acids are replaced by D-analogs, and they cannot be studied using high-resolution NMR and X-ray spectroscopy.

## 2. Results and Discussion

### 2.1. CIDNP Peculiarities in Chiral Linked Systems I–IV

**Scheme 1:**
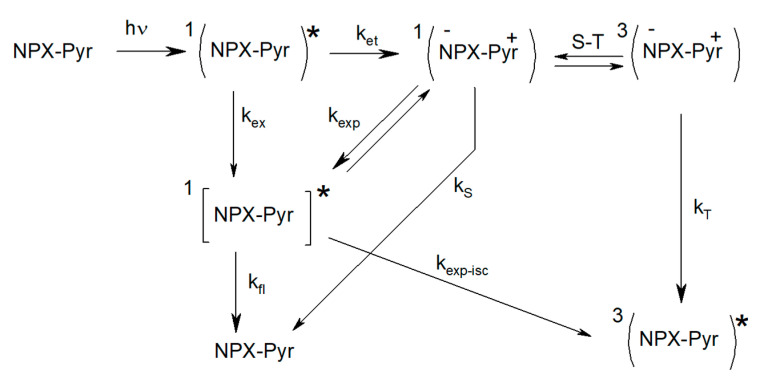
The path of the CIDNP formation under the UV irradiation of dyads I–II, where naproxen (NPX) linked with N-methylpyrrolidine (Pyr) [5]. The quantitative aspects of photoexcitation degradation are described in detail in Supplementary Materials (Scheme S1).

Below is a brief description of the processes that resulted in spin selectivity in chiral dyads. The consideration will begin with the most extensively studied dyads of naproxen with N-methylyrrolidine I and II (Figure 1). The scheme of CIDNP formation under the UV irradiation of these dyads is presented below (Scheme 1).

The ratios of the CIDNP coefficients of methyl protons in pyrrolidine fragment for diastereomers of dyads I and II, calculated taking into account the BZ concentration (*K*), are presented in Table 1.

Comparison of the experimental CIDNP effects with those calculated in the frame of the S-T_0_ approximation shows that, at a lifetime of 7 ns, which corresponds to the experimental data, significant hyperpolarization actually appeared only on N-CH_3_ protons and shows fivefold difference in the CIDNP values of N-CH_3_ and aromatic protons (the calculation results are presented in Supplementary Materials, Table S1). To trace the origin of spin selectivity, the hyperpolarization of protons in dyad II was calculated earlier in [10] using a two-position model with the Hamiltonian given in the introduction (1). Calculations have shown that the spin selectivity can be explained by the difference in the HFI constants in the BZ of the dyad’s diastereomers [10]. Qualitative explanations of the dependence of the spin selectivity on the solvent mixture are given in [10,11]. There, the dependence on the nature of the solvent is associated with the possibility of the formation of various associates that can change the BZ conformations and the spin density distribution, respectively.

Analysis of the nature of spin selectivity in the next two systems III and IV is more difficult due to following circumstances. First, the formation of exciplex being in fast dynamic equilibrium with BZ is not observed in these systems; therefore, we could not use its fluorescence lifetime as the BZ lifetime. Second, the presence of two chromophores in the dyads resulted in the appearance of an additional channel of excited state quenching—resonance singlet–singlet energy transfer (RET)—which reduces the accuracy of determining the BZ concentration. Third, in these systems, the difference in the HFI constants is much smaller than in dyads I and II; therefore, the calculation using one-nucleus approximation becomes impossible.

The next problem is that the CIDNP effects in dyads III and IV are observed on almost all nuclei and do not strictly correspond to the HFI of these nuclei. It should be emphasized that this inconsistency cannot be cleared up by varying the lifetimes of the BZ (the calculation of the values of CIDNP in the frame of S-T_0_ approximation for dyads III and IV are presented in Supplementary Materials, Tables S2 and S3). In addition, the (*R,S*)/(*S,S*) CIDNP ratios of these dyads somewhere differ for various protons.

**Scheme 2:**
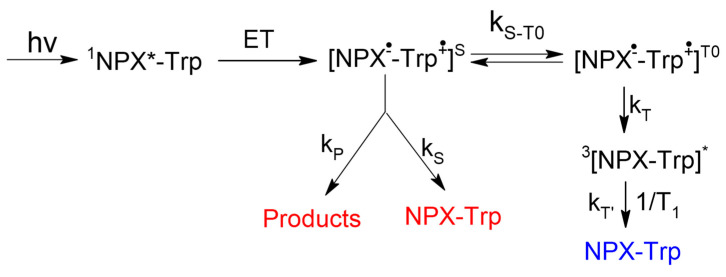
CIDNP formation under the UV irradiation of dyad III. Different signs of hyperpolarization are indicated by color.

The examples demonstrating the abovementioned features of the hyperpolarization and spin selectivity of dyads III and IV can be seen in the tables below. The mechanism of CIDNP formation in dyad III is presented in Scheme 2 [5].

All photophysical processes accompanying processes of ET and RET for dyads III and IV are shown in the schemes given in Supplementary Materials, Schemes S2 and S3.

As can be seen from Table 2, the inconsistencies between the CIDNP coefficients and the HFI constants vary from 17% for aromatic protons of KP to 4–7 times for protons of the tryptophan fragment.

In addition, the value of *K* for the 2-indole proton is almost two times different from the values of *K* for the other protons of the dyad III. Thus, this proton does not actually exhibit spin selectivity with decreasing polarity of the medium. If we take into account that the spin selectivity in the dyads was explained by the difference in the HFI constants in the BZ due to the difference of the diastereomer’s conformations, then the above results do not contradict this hypothesis. Indeed, the HFI constants in a linked system may differ from those in individual radicals, and the conformation may depend on solution polarity. A good example of such a dependency would be the proton in the second position of the indole ring, which can be H-bonded to the carboxyl moiety. The presence or absence of H-bond depends on the spatial arrangement of carboxyl fragment that can differ in diastereomers and, naturally, depends on the polarity of the medium. The values of spin selectivity of dyad III in solvents with different dielectric constants are listed in Table 3.

**Scheme 3:**
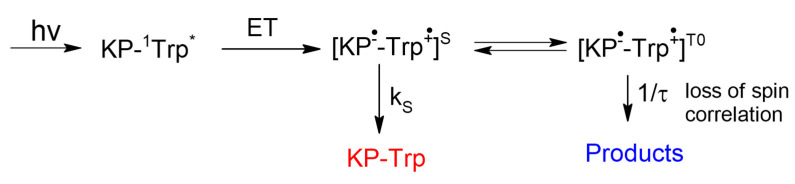
CIDNP formation under the UV irradiation of dyad IV [13,14].

Finally, as shown below, the latter system—dyad IV—on the contrary, demonstrates extreme differences between CIDNP of (*S,S*)-, (*R,S*)-, and (*S,R*)-optical isomers (see Table 4 and Table 5). First, we note that, for dyad IV, according to the arrangement of energy levels, in contrast to the dyads with NPX, the light also absorbs Trp, and the back electron transfer is possible only from the singlet spin state of the BZ (see Supplementary Materials, Scheme S3 and Scheme 3).

Despite this, described in the literature, dyads with KP, where donors are N-methylpyrrolidine, cholesterol, and tryptophan, demonstrate CIDNP effects with different signs for the initial dyad and products [8,9]. Since the escape of radicals into the bulk is impossible in a linked system without its cleavage, we assume that the appearance of different signal signs of the initial dyads and products is the result of the loss of spin correlation. It occurs in the triplet state of the biradicals.

In this system, the differences in the ratios of the constants of various protons and the CIDNP coefficients vary from two to ten (Table 4). In addition, the protons in the second position of the indole ring in the solution with high polarity demonstrate the maximum difference for (*S,S*)- and (*R,S*)-, (*S,R*)-isomers, in contrast to the dyad with NPX (compare Table 3 with Table 4 and Table 5). Such difference in the behavior of the proton in 2-position of the indole ring in dyads with NPX and KP can be associated with different positions of the carboxyl group relative to this proton in different conformations of two dyads.

In conclusion of this part, it should be noted that the consideration of biradicals in the linked systems as two separate radicals of donor and acceptor is unlikely to be correct. In addition to this work, the comparison of hyperpolarization with the calculated HFI constants for individual components of the triad was carried out in the work [18]. In this case, violations of proportionality between the values of hyperpolarization and HFI constants were also observed.

However, in our case, there is one more reason for discussed inconsistency of CIDNP with HFI constants, in addition to the abovementioned differences in BZ conformations. These discrepancies can be associated with the fact that ET occurs in the dimers, and accordingly, BZ is a part of dimer formed through an H-bond between NH-C=O fragments of two molecules or between the NH group of one molecule and ether group of another [10]. This leads to the additional change in the BZ conformations as compared to free BZ, and in turn, spin density distribution in BZ is changed. Data indicating that, for dyads II and III, ET occurs in dimers was presented in works [10,11]. In the next section, we explore this possibility for diastereomers of dyad IV.

### 2.2. Dependence of CIDNP Enhancement Coefficient on the Concentration Ratio of Diastereomers of Dyad IV

**Scheme 4:**
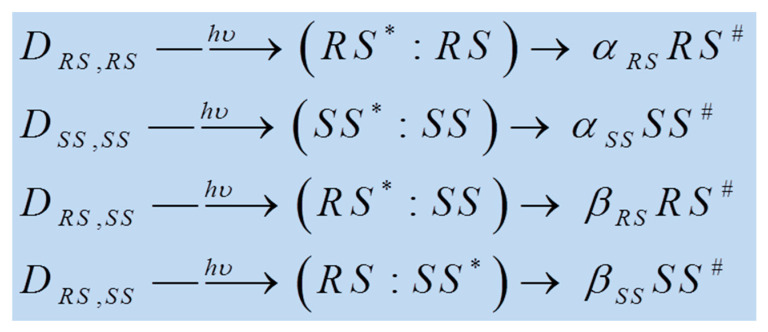
Photoinduced processes occurring in homo-(*D_RS,RS_, D_SS,SS_*) and hetero-*D_RS,SS_* chiral dyad dimers; α and β—CIDNP enhancement coefficient, α >> β; *RS*^#^, *SS*^#^—polarized dyads.

The presented evidence of the role of dimers in the photoinduced ET in dyads is based on Frank’s theory, which is well known [11]. According to this theory, chiral systems tend to associate and at the same time catalyze the formation of their own kind. In our case, it means that the following types of dimers can be formed: homo ((*S,S*–*S,S*), (*S,R*–*S,R*), and (*R,S*–*R,S*)) and hetero ((*S,S*–*S,R*), (*S,S*–*R,S*), and (*R,S*–*S,R*)). However, the last configuration most likely should be attributed to homo dimers, since the (*S,R*)- and (*R,S*)-configurations are enantiomers. Then, according to the hypothesis formulated in [10], the efficiency of CIDNP formed upon UV irradiation of homo dimers should be much higher than that for hetero dimers (Scheme 4). This assumption was investigated in detail and was fully confirmed by the results of CIDNP dependence on the concentration ratios of (*S,S*)- and (*R,S*)-II upon UV-irradiation of the mixtures of diastereomers [10,11].

**Figure 2:**
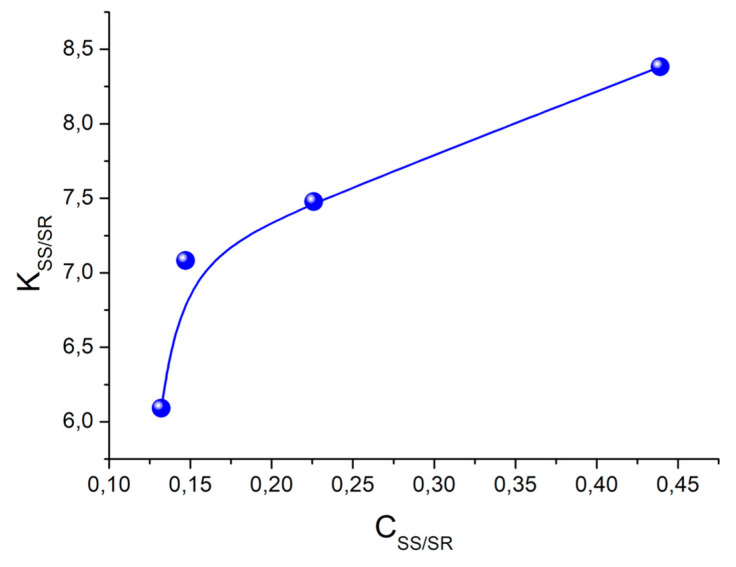
Dependence of the ratio of the CIDNP enhancement coefficient (*K*) on the concentration ratio of diastereomers of dyad IV.

To trace the applicability of this hypothesis to various configurations of dyad IV, a series of experiments with UV irradiation of different mixtures of diastereomers was carried out. Sequential addition of (*S,S*)- or (*S,R*)- to the mixture of (*R,S*)- and (*S,S*)-diastereomers gave the following results. Figure 2 clearly shows that, in accordance with the Scheme 4, the ratio of the CIDNP enhancement coefficients (*SS/RS*) increases with an increase in the (*S,S*) contribution to the diastereomers concentration ratio.

The behavior of the CIDNP coefficients for both (*S,S*)- and (*R,S*)-diastereomers can also be traced by analyzing the data from Table 6. Initially weak hyperpolarization of (*R,S*)- was found to decrease with an increase in its own concentration. Moreover, CIDNP of (*R,S*)- increases with the addition of (*S,S*)-, and these two facts let us assume the formation of different types of associates in the case of (*S,S*)- and (*R,S*)-configurations. It can be assumed that (*R,S*)- associates are larger than dimers, and then the addition of (*S,S*) can change their structures, leading to a smaller particles. Differences in the CIDNP coefficients in large and small associates can, first of all, arise as a consequence of the eliminating hyperpolarization by magnetic dipole–dipole interaction of electrons, which is averaged or not averaged depending on the particle size. It should be noted that, at the highest (*S,S*)- concentrations, its CIDNP coefficient also decreases (bottom line of Table 6). This may be due to the enlargement of the associates. The physical reasons for the suppression of polarization generated in large particles are explained in detail in Section 2.4.

At the same time, the formation of associates can in no way explain the five- to tenfold differences in the CIDNP values of diastereomers of dyad IV by the change in their HFI constants in BZ. Therefore, further attempts will be made to reveal possible structural differences in the (*S,S*)-, (*R,S*)-, and (*S,R*)-optical isomers of dyad IV, as well as their tendency to form associates using molecular dynamics and quantum chemical calculations.

### 2.3. Molecular Dynamics Simulations

**Figure 3:**
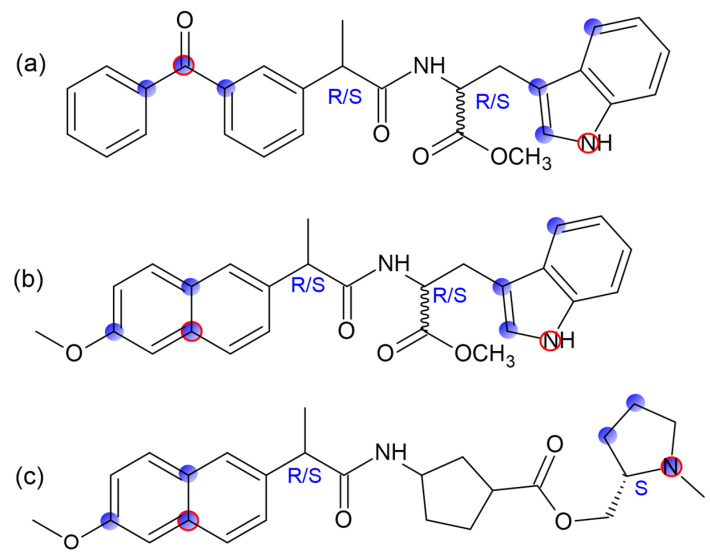
Selection of atoms for distance calculation (red circles) and planes for angle calculation (blue circles) in dyad (**a**) IV, (**b**) III, and (**c**) II.

Further, we tried to trace the differences between optical configurations of dyad IV using molecular modeling and compare with the results obtained for other dyads: II and III. The molecular dynamics (MD) simulations were performed using GROMACS 2018.4 package [19,20,21,22]. The mean time of contact of donor and acceptor sites (at the distance less than 0.6 nm and less than 0.45 nm) and the mean angle between the planes of the donor and acceptor residues were calculated. The atom selection for each dyad is given in Figure 3. The results of the analysis of MD trajectories are shown in Table 7.

**Figure 4:**
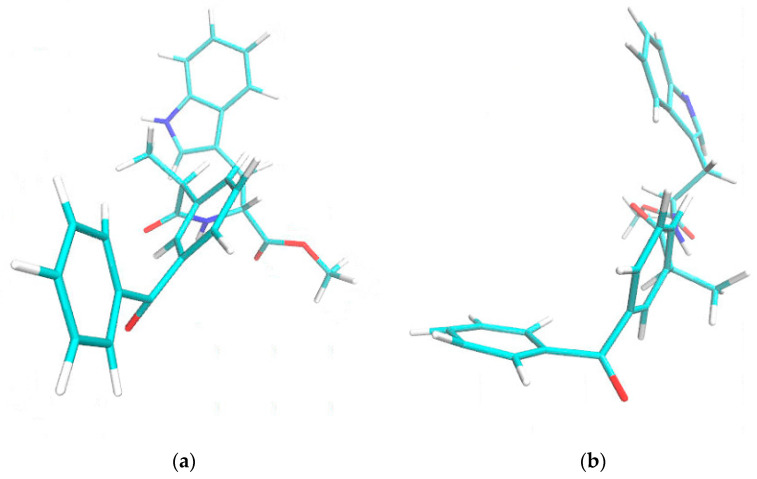
Snapshots of MD trajectories for (**a**) (*S,R*)-IV and (**b**) (*R,S*)-IV illustrating different mutual orientation of ketoprofen aromatic rings.

It is noticeable that (*S,R*)- and (*R,S*)-enantiomers of dyad IV have significantly different mutual orientation of ketoprofen aromatic rings (Figure 4). The mean angle between planes of ketoprofen aromatic rings is 88° ± 1° for (*R,S*)-enantiomer, 90° ± 0.5° for (*S,S*)-enantiomer, and 49° ± 0.5° for (*S,R*)-enantiomer.

**Figure 5:**
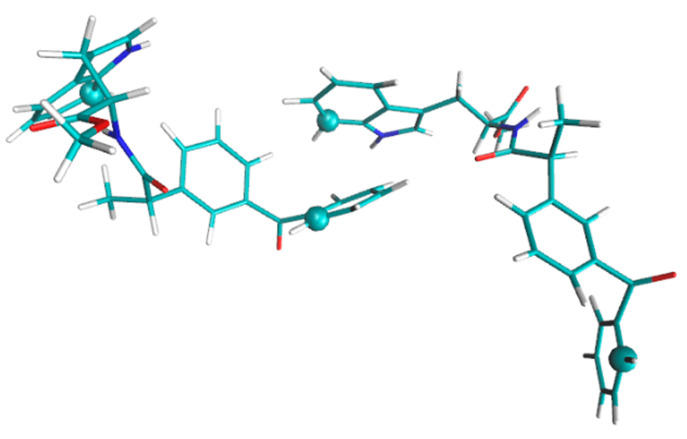
Example of dimer structures for (*R,S*)-IV. The spheres mark the atoms, the distance between which was taken for analysis.

**Figure 6:**
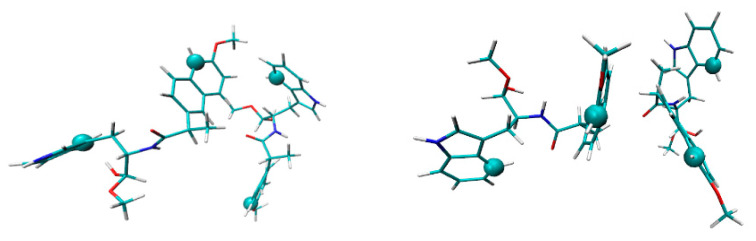
Dimer configurations for the (*S,S*)-III. The case of the closest arrangement of a naproxen fragment with a tryptophan (**left**) and two naproxen residues (**right**). The spheres mark the atoms, the distance between which was taken for analysis.

The same simulations were performed for (*R,S*)- and (*S,S*)-III. Atom choices for distance and angle calculations are illustrated at Figure 3b. The following are the results of the evaluation of the possibility of dimer formation in optical isomers of the dyads IV and III (Figure 5 and Figure 6).

Table 8 below shows the fraction of time (%) with which the diastereomers of the dyads IV and III exist in the form of dimers.

From the data in Table 8, the probability of dimer formation for the (*S,S*)-configuration was always less than for the (*R,S*)-analog. The higher stability of the (*S,S*)-configuration as compared with the (*R,S*)-configuration is also indicated by the described below data for quantum chemical simulation of various optical configurations of dyad IV.

### 2.4. Quantum Chemical Simulation of Various Optical Configurations of Dyad IV

**Figure 7:**
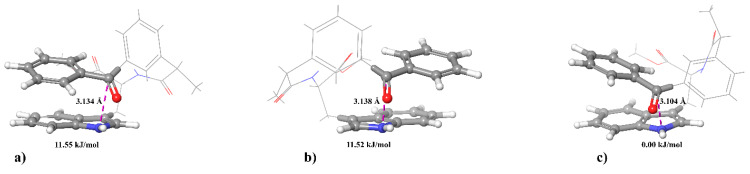
Structural parameters of optical isomers (**a**) (*R,S*)-; (**b**) (*S,R*)-; (**c**) (*S,S*)- of dyad IV: Gibb’s energy values calculated under solvent conditions (acetonitrile, implicit solvation). Values in the gas phase are presented in Materials and Methods.

In addition to the above, another attempt was made; this time, this is a quantum-chemical simulation of the structure and properties of various optical configurations of the dyad IV. According to the calculation results (Figure 7), (*S,S*)-isomer has the lowest energy; (*S,R*)- and (*R,S*)-isomers, respectively, make up almost the same difference in the values of 11.52 and 11.55 kJ (in acetonitrile). Potentially important distances between the nitrogen atom of the tryptophan moiety and the carbon atom of the ketoprofen moiety were also measured.

Next, in Section 2.5, we use the obtained distances to calculate the hypepolarization in optical isomers of dyad IV using the newly developed theory.

### 2.5. Calculation of CIDNP Effects for Dyads III, IV within S-T_0_ Approximation

Further, an effort was made to use the obtained data to calculate hyperpolarization using simulation times as analogs of the BZ lifetimes. As mentioned above, we have experimental data on the lifetimes of BZ diastereomers only for dyads I–II. Therefore, it was of interest to compare the spin selectivity for all systems within the framework of a unified approach: to calculate CIDNP effects using molecular modeling data. As can be seen from the data in Table 9, calculated CIDNP of the dyads with naproxen show the results coinciding with the experimental ones. As for dyad IV, even the use of different distances, along with different lifetimes, for the (*S,S*)- and (*S,R*)-diastereomers does not lead to a tenfold difference in the values of hyperpolarization.

Thus, we conclude that it is not possible to describe the extremely low hyperpolarization of the (*R,S*)- and (*S,R*)-configurations of dyad IV in the frame of classical radical-pair theory. At the same time, MD simulating and CIDNP calculation in the model processes in short linked systems really shows the differences in the dyads with L- and D-tryptophan: greater stability of the (*S,S*)-configuration in comparison with other analogs, different probability of dimer formation, and different distribution of spin density (spin selectivity) for (*S,S*)- and (*R,S*)-configuration.

### 2.6. Influence of Electron Magnetic Dipole–Dipole Interaction on the CIDNP Efficacy. The Investigation Using Two-Position Model

To explain the anomalously low hyperpolarization of the (*S,R*)- and (*R,S*)-optical configurations of dyad IV, we used a modified version of the RP theory, including the magnetic dipole–dipole interaction of electrons within the framework of the two-position model. The description of the two-position model can be found everywhere (for example, [10,23]). The following approximations were used: the HFI constants (a_1_ = a_2_) are the same for both partners of the BZ, the electron–electron, dipole–dipole interaction (A) is included in both in the reaction zone and outside it. The angle of precession of magnetic dipole around external magnetic field is indicated by Ѳ.

**Figure 8:**
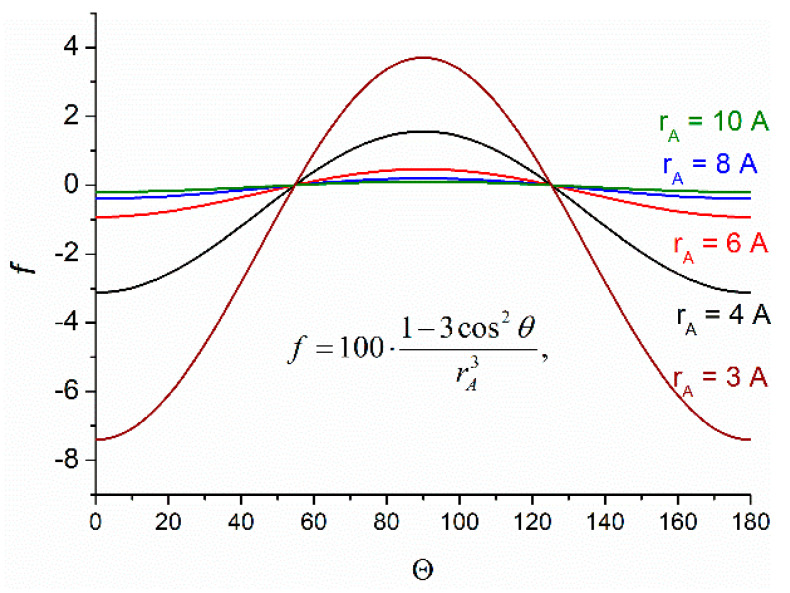
Dependence of the electron dipole–dipole interaction on the angle θ between radius vector connecting the paramagnetic centers and the direction of the external magnetic field.

Expressions that determine the polarization in the optical isomers of dyad IV are described in Supplementary Materials (Section S3). Below is a qualitative analysis of the obtained expressions. Figure 8 demonstrates the dependence of electron dipole–dipole interaction (A) on θ at different distances between paramagnetic centers in BZ.

**Figure 9:**
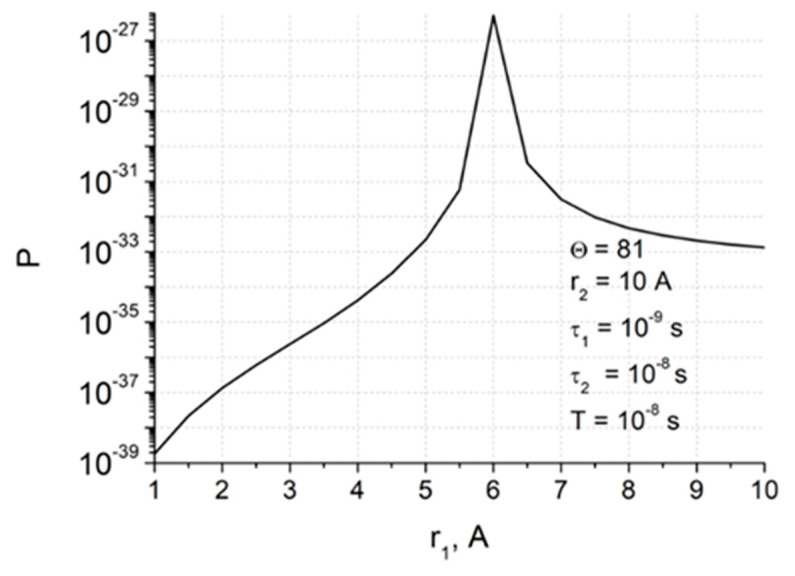
Dependence of hyperpolarization (P) on the distance between paramagnetic centers in the reaction zone.

Analysis of the dependences of hyperpolarization value (P) on θ at different distances in the reaction zone (r_1_ = 3 A and 4 A) demonstrate complete elimination of hyperpolarization at any used residence times of the system in the reaction zone. For clarity, below is shown the dependence of hyperpolarization on the distance in the reaction zone at fixed angles θ, distance out of the reaction zone, and lifetimes in these zones (Figure 9).

Comparison of the values of hyperpolarization in Table 9 and Figure 9 shows that the differences are tens of orders of magnitude. Therefore, if the magnetic dipole–dipole interaction is not averaged, as it usually happens in the liquid phase, then it has to completely suppress hyperpolarization.

Now it is necessary to understand what is the reason for the differences in the (*S,R*)- and (*R,S*)-configuration of the dyad IV compared to the (*S,S*)-diastereomer of this dyad. Within the framework of the approach used, the characteristic quantity that determines whether the magnetic dipole–dipole interaction of electrons is averaged is the product of the amplitude of the magnetic dipole–dipole interaction (A) by the characteristic time of rotational correlation (τ_c_). When the value of this product is much greater than 1, the dipole–dipole interaction is averaged. An estimate of the corresponding characteristic correlation time at an amplitude of the dipole–dipole interaction of 6.068 × 10^9^ in frequency units (at r_a_ = 3 A) gives a value of 0.16 ns. Further estimation of the characteristic particle size at which the dipole–dipole interaction is averaged was carried out using the following formula:

(3) $$\tau_{c}=\frac{1}{2D}=\frac{3\eta V}{k_{B}T}=\frac{4\pi \eta R^{3}}{k_{B}T}$$

where *τ_c_* is the characteristic time of rotational diffusion, *D* is the rotational diffusion coefficient, *η* is the viscosity of acetonitrile (0.35 × 10^−3^ Pa·s), *k_B_* is the Boltzmann constant, and V and R are the volume and radius of the particle. As a result, when the particle size is much larger than 5.5 A, the dipole–dipole interaction is not averaged.

If we take into account that the MD simulation shows a higher probability of dimer formation for the (*R,S*)-configuration as compared to the (*S,S*)-configuration (Figure 5) and our experimental data from Section 2.3 confirm this, then the differences in the volumes of particles formed from (*S,S*)-, (*R,S*)-, and (*S,R*)-configuration are obvious.

As for other differences between (*S,S*)- and (*R,S*)-diastereomers of dyad IV, it follows from the data of quantum chemical calculations that (*S,S*)- is more stable and has a larger energy barrier for the transition between conformations than (*R,S*)- [18]. We can only assume that the associates of (*S,R*)- and (*R,S*)-configurations of dyad IV have a more complex structure, in which there is no complete averaging of the electron dipole–dipole interaction. Molecular modeling data let us suggest associations involving both the ketoprofen moiety and the NH bonds of enantiomers. After all, it is known that the appearance of even one D-isomer changes the structures of many proteins and peptides [2].

## 3. Materials and Methods

### 3.1. Synthesis

The synthesis of dyad (*S,R*)-III with D-Trp was performed according to a method described earlier [24]. The synthesis of other dyads, I, II, and IV, was described in [14,25]. An Agilent 1260 Infinity HPLC technique with a diode array detector was used for purification and analysis of purity of individual isomers. A semi-preparative column (Diaspher-110-C18, 10 × 250 mm, 5 µm, BioChemMack ST Ltd., Moscow, Russia) was used for purification of individual isomers. The purification was carried out using a mobile phase composed of acetonitrile (ChimMed, Moscow, Russia) and water (50:50) at a flow rate of 4 mL/min with detection at 260 nm. The injection volumes were 250 µL. The purity of isolated isomers was analyzed on a C18 column (Diaspher110-C18, 2 × 120 mm, 5 µm, BioChemMack ST Ltd., Moscow, Russia). The analyses were carried out using a mobile phase composed of acetonitrile and water (38:62) at a flow rate of 0.7 mL/min with detection at 260 nm. The injection volume was 5 µL. The purities of individual isomers isolated from racemate mixture were 99.9% for (*R,S*) and (*S,S*), respectively.

### 3.2. NMR and Photo-CIDNP Measurements

^1^H NMR and photo-CIDNP experiments were performed on a DPX-200 NMR spectrometer (Bruker, Germany, 200 MHz ^1^H operating frequency, P(π/2) = 2.5 µs). A Lambda Physik EMG 101 MSC eximer laser was used as a light source (308 nm, 100 mJ at output window, 20 mJ/pulse in sample volume, pulse duration 15 ns) in the CIDNP experiments. The samples in standard 5 mm Pyrex NMR tubes were irradiated directly in the NMR probe of DPX-200 NMR spectrometer. The samples were bubbled with argon for 15 min to remove dissolved oxygen just before photolysis. Acetonitrile-d3 (Aldrich, D 99.8%, St. Louis, MO, USA) was used as solvent.

**Figure 10:**
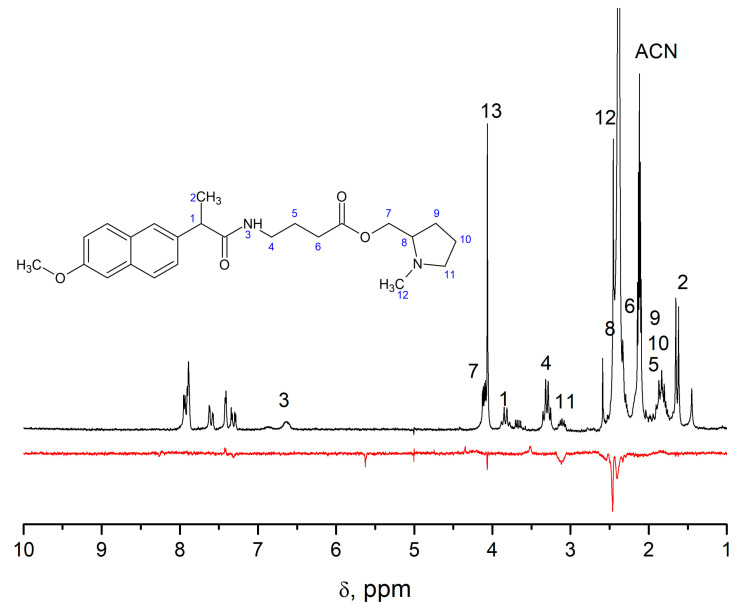
^1^H NMR (black) and CIDNP spectra (red) of 4 mM (*R,S*)-I in CD_3_CN. NMR spectra of (*R,S*)- and (*S,S*)-I are identical.

**Figure 11:**
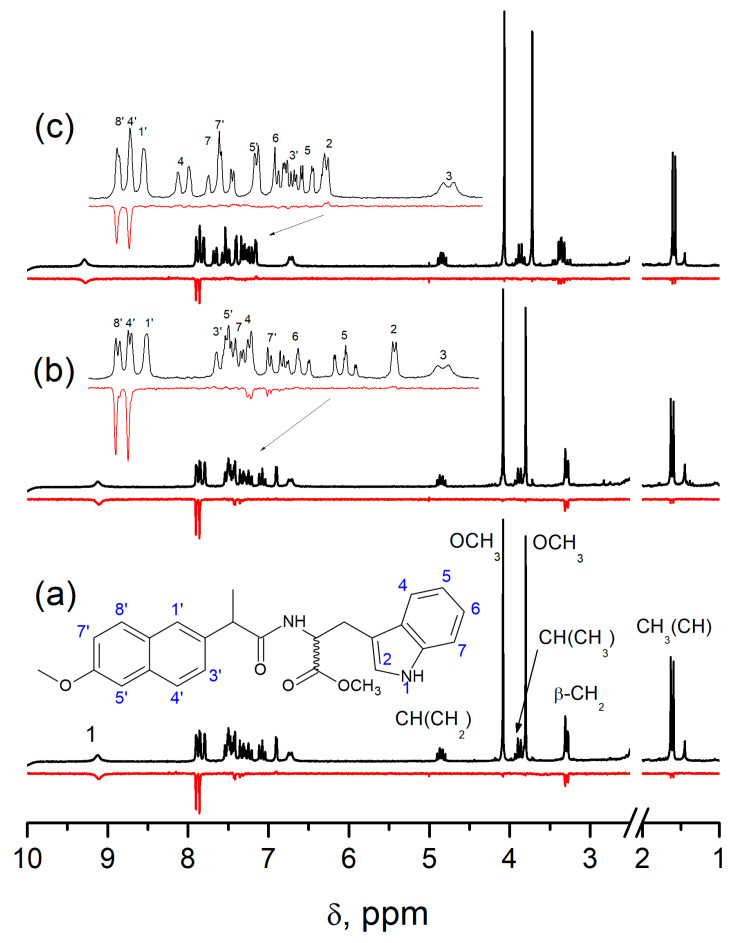
^1^H NMR (black) and CIDNP spectra (red) of 5 mM solutions of (**a**) (*R,S*)-III, (**b**) (*S,R*)-III, and (**c**) (*S,S*)-III in CD_3_CN. Inserts show the expanded region of resonance for aromatic protons.

**Figure 12:**
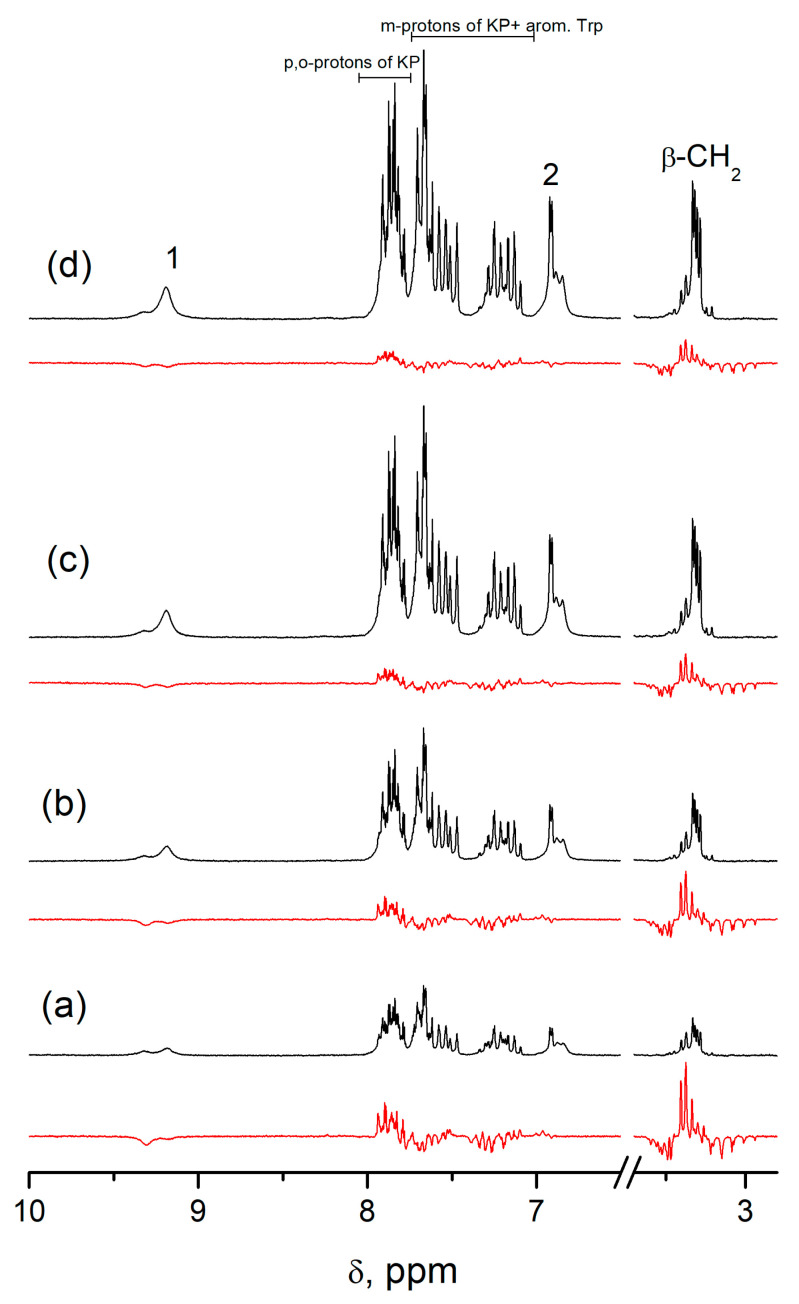
^1^H NMR (black) and CIDNP spectra (red) of diastereomers mixtures of dyad IV with the following concentrations: (**a**) 1.8 mM (*S,S*)+ 4.1 mM (*R,S*); (**b**) 2.1 mM (*S,S*)+ 9.3 mM (*R,S*); (**c**) 2.6 mM (*S,S*)+ 17.7 mM (*R,S*); (**d**) 2.8 mM (*S,S*)+ 21.2 mM (*R,S*) in CD_3_CN.

The photo-CIDNP was performed as pseudo steady state (PSS) experiments. To carry it out, a standard pulse sequence was used: presaturation; delay 1; pulse τ(π); delay2 (16 laser flashes with repetition rate 50 Hz during delay2); observation pulse τ(π/2); acquisition. Delay1/delay2 ≈ 1.1 to remove residual signals of solvents and solutes. After laser irradiation, the ^1^H NMR spectra of products were recorded. Further, the typical ^1^H NMR and CIDNP spectra of dyads I, III, and IV in acetonitrile-d_3_ are presented (Figure 10, Figure 11 and Figure 12).

^1^H NMR and CIDNP spectra of three optical configurations (*R,S*)-, (*S,R*)-, and (*S,S*)- of dyad III are pictured below. Note that NMR and CIDNP spectra for (*R,S*)- and (*S,R*)-enantiomers are the same (Figure 11a,b).

Differences on the chemical shifts of NH (1) protons in diastereomers of dyad IV allowed us to study CIDNP effects in the mixture of two diastereomers (Figure 12).

Absorption coefficients of diastereomers are the same, and at 308 nm, is equal to 200 ± 20 M^−1^ cm^−1^. Therefore, optical density in 5 mm Pyrex NMR tube was varied from ca. 0.4 to 2.4.

### 3.3. Calculation of CIDNP Effects within S-T_0_ Approximation

Calculation of the CIDNP effects at high magnetic fields was carried out according to the Adrian model within the framework of the S-T_0_ approximation. A program developed by [26] and modified in [27] was used. Calculation parameters: corresponding HFI constants listed in Table 1, Table 2 and Table 4; Δg (I, II, IV) = 6 × 10^−4^, Δg (III) = 4 × 10^−4^; magnetic field 47,000 G; diffusion coefficient D = 10^−6^ cm^2^/s; Onsager radius 15 A.

### 3.4. Molecular Dynamic Simulations

The molecular dynamics simulations were performed using the GROMACS 2018.4 package and GROMOS54a7 force field [28,29]. The topology of dyads was built using the Automated Topology Builder [19]. The simulation was performed in the NPT ensemble with constant pressure (1 bar) and constant temperature T = 300 K, which were maintained by the semiisotropic Parrinello–Rahman barostat [20]. For electrostatic interactions, PME method with the fourth-order of cubic interpolation and the grid of 0.16 was used [21,22]. Size of model boxes was approximately 6 × 6 × 6 nm. Model box contained 1 or 2 dyad molecules and ~2300 acetonitrile molecules (~13,700 atoms). For each system, 10 MD trajectories with the duration of 100 ns were obtained. Using the standard GROMACS utilities, the time dependence of the distance between the marked atoms (Figure 3a) was obtained. Further, the average time spent by these groups at a distance of less than 0.6 nm and less than 0.45 nm from each other was calculated. It should be noted that the figures were obtained for launches with a length of 100 ns, i.e., it is more correct to correlate the results with the fraction of time that the atoms are at a distance of less than 0.6 nm.

### 3.5. Quantum-Chemical Calculation

The optical isomers of dyad IV have been studied using quantum chemical modeling. The first step was conformational analysis in Jaguar software (QM Conformer and Tautomer Predictor) [30] for each of the studied systems. Next, the most suitable conformers with minimal distances between the nitrogen atom of the tryptophan fragment and the carbon atom of the ketoprofen fragment were taken. The next step was the implicit accounting optimization (PCM) [31] of acetonitrile using the Jaguar software [30] using the M06-2X-D3/cc-pVTZ (-f) method [32,33].

According to Table 10, (*S,S*)-isomer has the lowest energy whereas (*S,R*)- and (*R,S*)-isomers have the same difference in Gibb’s energy values calculated under solvent conditions (acetonitrile, implicit solvation). Concerning distances between the nitrogen atom of the tryptophan moiety and the carbon atom of ketoprofen, there are no significant differences between the isomers (Table 11). Molecular structures of dyad IV isomers in Cartesian coordinates (XYZ-files) are presented in Supplementary Materials (Section S4).

## 4. Conclusions

An analysis of spin effects (hyperpolarization and spin selectivity) in donor–acceptor dyads and a comparison of the experimental results with MD calculations allowed us to draw some conclusions about the influence of the optical configuration of the dyads on their structure and reactivity. First of all, this is the high sensitivity of spin effects to changes in the chiral configuration. So, it has been demonstrated that, in all studied dyads, the hyperpolarization intensities of all protons change when the configuration of only one chiral center changes. This is an important result in light of the fact that a change in the configuration of even one amino acid leads to irreversible changes in the structure of many proteins and peptides. The discrepancy between the CIDNP coefficients and the HFI constants of the dyad partners indicates that the HFI constants in the BZ do not always coincide with the constants of individual radicals. This can be either the roughness of the used approximation or the result of a difference in the conformations of BZ diastereomers. If ET in dyads occurs in dimers, as shown earlier and further confirmed in this paper, then difference in conformations of different configuration seems even more likely. The dimers formation was also confirmed by the MD calculations. In addition, the results of MD showed that the (*S,S*)-configuration of the dyads is the most stable. As for the (*R,S*) and (*S,R*)-enantiomers of dyad IV, it is shown that the hyperpolarization arising in the process of ET in this system does not described within the framework of the classical theory of radical pairs. To describe it, a special approach was developed that includes the electron–electron magnetic dipole–dipole interaction in the secular equation of spin chemistry.

## Figures and Tables

**Table 1:** Spin selectivity: *RS/SS* ratio of CIDNP coefficients (*K*) of protons (marked with red circles) for diastereomers of dyad I and corresponding HFI constants (G) in BZ (top) [15]; the same for dyad II (below, taken from [10]).

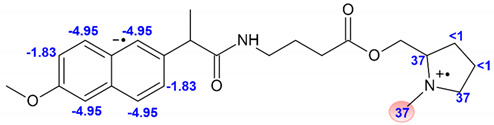	
%ACN	K_RS/SS_ (N-CH_3_)
100	1.3 ± 0.1
** 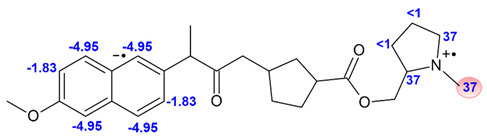 **	
%ACN	K_RS/SS_ (N-CH_3_)
100	1.5 ± 0.2
80	2.1 ± 0.2
60	1.9 ± 0.2

**Table 2:** The ratios of HFI constants and the observed CIDNP coefficients (*K*) relative to 1-NH and the values of the observed CIDNP coefficients for protons of dyad III in CD_3_CN solution.

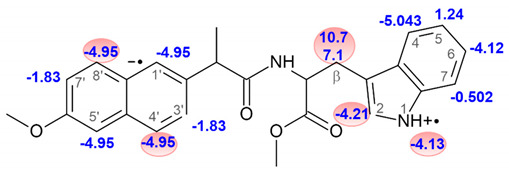				
Position	8′/4′	β-CH_2_	2	1
HFI_i_/HFI_(1)_	1.2	2.6–1.7	1.02	1
*K_i_/K_(1)_*	1.4–1.1	0.27–0.24	0.064–0.021	1
*K_RS_*	1.4 ± 0.2	0.27 ± 0.03	0.064 ± 0.07	1.0 ± 0.1
*K_SR_*	1.4 ± 0.2	0.31 ± 0.03	0.025 ± 0.006	1.14 ± 0.07
*K_SS_*	0.76 ± 0.02	0.16 ± 0.02	0.038 ± 0.003	0.68 ± 0.01
*K_RS/SS_*	1.8 ± 0.2	1.7 ± 0.2	1.7 ± 0.2	1.5 ± 0.2

**Table 3:** Spin selectivity of protons in diastereomers of dyad III detected in solvent with different dielectric constants (mixtures of CD_3_CN/C_6_D_6_).

Volume Fraction of CD_3_CN, %	Ratio of CIDNP Coefficients (*K_RS/SS_*) of Diastereomers for Different Protons			
	8′/4′	β-CH_2_	2	1
100	1.8 ± 0.2	1.7 ± 0.2	1.7 ± 0.2	1.5 ± 0.2
80	1.9 ± 0.2	2.2 ± 0.2	1.3 ± 0.1	-
60	1.9 ± 0.2	2.0 ± 0.2	0.9 ± 0.1	-
40	2.0 ± 0.2	1.7 ± 0.2	1.0 ± 0.1	1.8 ± 0.2
20	1.6 ± 0.2	1.8 ± 0.2	1.1 ± 0.1	1.6 ± 0.2
0	1.5 ± 0.2	-	-	2.0 ± 0.2

**Table 4:** The ratios of HFI constants and observed CIDNP coefficients (*K*) relative to 1-NH and the values of observed CIDNP coefficients for protons of dyad IV diastereomers in CD_3_CN.

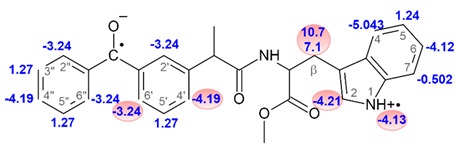				
Position	4′/6′	β-CH_2_	2	1
HFI_i_/HFI_(1)_ *	0.90	2.6–1.7	1.02	1
*K_i_/K_(1)_*	0.6–0.5	1.4–1.3	0.3–0.1	1
*K_SS_*	1.4 ± 0.1	3.0 ± 0.3	0.6 ± 0.1	2.2 ± 0.2
*K_SR_*	0.11 ± 0.01	0.3 ± 0.1	0.03 ± 0.01	0.21 ± 0.02
*K_SS/SR_*	13 ± 1	10 ± 1	20 ± 2	10 ± 1

* HFI constants are taken from [16,17,18].

**Table 5:** Spin selectivity of protons in diastereomers of dyad IV detected in solvent with different dielectric constants (CD_3_CN/C_6_D_6_ mixtures).

Volume Fraction of CD_3_CN, %	Ratio of CIDNP Coefficients (*K_SS/SR_*) of Diastereomers for Different Protons			
	4′/6′	β-CH_2_	2	1
100	13 ± 1	10 ± 1	20 ± 2	10 ± 1
80	11 ± 1	9.3 ± 0.9	13 ± 2	8.9 ± 0.9
60	11 ± 1	9.2 ± 0.9	15.0 ± 2	9.4 ± 0.9
40	6.5 ± 0.7	*	5.6 ± 0.6	9.0 ± 0.9
20	9.3 ± 0.9	*	11 ± 1	7.1 ± 0.7

* The data are not presented due to changes occurring in the structure of the multiplet with a change of the solution’s mixture. The latter reduces the integration accuracy.

**Table 6:** Dependence of CIDNP coefficient (*K*) on the concentration of (*R,S*)- and (*S,S*)-diastereomers. The initial concentrations in the first experiment are 1.2 × 10^−3^ M of (*S,S*) and 3.6 × 10^−3^ M of (*R,S*), in the second—0.9 × 10^−3^ M of (*S,S*) and 5.0 × 10^−3^ M of (*R,S*). Subsequent additions of (*S,R*)- or (*S,S*)- are indicated as the sum of initial concentrations and the additive in the columns C*_SR_* and C*_SS_*. Optical density was varied from ca. 0.5 to 1.0.

	C*_SS_*, mM	*K_SS_*	C*_RS_*, mM	*K_RS_*
Addition of (*S,R*)- into the (*S,S*)+(*R,S*) mixture	1.2	4.00	3.6	0.64
	1.2	3.20	5.8	0.43
	1.3	2.70	9.2	0.31
Addition of (*S,S*)- into the (*S,S*)+(*R,S*) mixture	0.9	2.16	5.0	0.23
	3.5	2.24	5.2	0.28
	5.7	1.95	5.4	0.25

**Table 7:** Times of being at a certain distance of (*R,S*)-, (*S,R*)-, and (*S,S*)-configuration of dyads IV–II and angles between plane of donors and acceptors in the solution of acetonitrile (mean values from 10 trajectories with the duration of 100 ns).

	Time (<0.6 nm)	Angle (<0.6 nm)	Time (<0.45 nm)	Angle (<0.45 nm)
(*R,S*)-IV	4.4 ± 0.6 ns	91° ± 4°	1 ± 0.1 ns	81° ± 2°
(*S,R*)-IV	7 ± 0.4 ns	79° ± 5°	0.8 ± 0.1 ns	89° ± 1°
(*S,S*)-IV	7.4 ± 0.3 ns	92° ± 1°	1.5 ± 0.1 ns	105° ± 2°
(*R,S*)-III	35 ± 2 ns	77.5° ± 1°	12.3 ± 0.8 ns	95° ± 1°
(*S,S*)-III	12 ± 1.5 ns	77.5° ± 1.5°	5.1 ± 0.5 ns	89° ± 1.5°
(*R,S*)-II	0.2 ± 0.1 ns	84° ± 3°	<0.01 ns	-
(*S,S*)-II	0.05 ± 0.02 ns	74° ± 6°	<0.01 ns	-

**Table 8:** The comparison of the possibility of dimer formation for various optical configurations of dyads IV and III.

	The Fraction of Time Spent in Dimer Form, %
(*R,S*)-III	0.81 ± 0.08
(*S,S*)-III	0.46 ± 0.05
(*R,S*)-IV	0.6 ± 0.03
(*S,S*)-IV	0.38 ± 0.04

**Table 9:** Calculated CIDNP effects for dyads III, IV within S-T_0_ approximation using times analogs from MD simulations.

Dyad	CIDNP for Different Protons, a.u.							Time, ns	Contact Radius, A
	6′	4′	β-CH_2_	2	1	4	6		
(*S,R*)-IV	2.4 × 10^−3^	3.2 × 10^−3^	6.9 × 10^−3^	−3.2 × 10^−3^	−3.1 × 10^−3^	−3.9 × 10^−3^	−3.1 × 10^−3^	1	4.5
(*S,S*)-IV	1.7 × 10^−3^	2.3 × 10^−3^	5.0 × 10^−3^	−2.3 × 10^−3^	−2.3 × 10^−3^	−2.9 × 10^−3^	−2.3 × 10^−3^	7.4	6.0
	4′		β-CH_2_	2	1	4	6		
(*S,S*)-III	−4.2 × 10^−3^		−8.4 × 10^−3^	3.3 × 10^−3^	3.1 × 10^−3^	4.1 × 10^−3^	3.2 × 10^−3^	12	7.0
(*R,S*)-III	−2.4 × 10^−3^		−4.9 × 10^−3^	1.9 × 10^−3^	1.7 × 10^−3^	2.4 × 10^−3^	1.7 × 10^−3^	35	7.0

**Table 10:** Energy characteristics of (*R,S*)-, (*S,R*)-, and (*S,S*)-optical isomers IV.

Compound	G_gas_, Hartree	G_gas_, kJ	G_sol_, Hartree	G_sol_, kJ	HOMO-LUMO Gap
(*R,S*)-IV	−1492.989906	8.89	−1493.012489	11.55	−0.21920
(*S,R*)-IV	−1492.990052	8.51	−1493.012502	11.52	−0.21922
(*S,S*)-IV	−1492.993292	0.00	−1493.016890	0.00	−0.21461

**Table 11:** Distances between the nitrogen atom of the tryptophan fragment and the carbon atom of the ketoprofen fragment.

Compound	*_d_*_N-C, Å_
(*R,S*)-IV	3.134
(*S,R*)-IV	3.138
(*S,S*)-IV	3.104

## Supplementary Materials

The following are available online at https://www.mdpi.com/article/10.3390/ijms23010038/s1.
