# Supplementary material for: Optical Configuration Effect on the Structure and Reactivity of Diastereomers Revealed by Spin Effects and Molecular Dynamics Calculations
Source: Int J Mol Sci. 2021 Dec 21;23(1):38. doi: 10.3390/ijms23010038 (PMC8744724; doi:10.3390/ijms23010038)
Supplement: Supplementary file 1 [file ijms-23-00038-s001.zip › ijms-1516305-supplementary.pdf]

SUPPLEMENTARY MATERIALS

Optical configuration effect on the structure and reactivity of diastereomers revealed by spin effects and molecular dynamics calculations

Aleksandra A. Ageeva[1,2], Alexander B. Doktorov[1,*], Olga Yu. Selyutina[1], Ilya M. Magin[1,2], Margarita G. Ilyina[3], Sophia S. Borisevich[3,4], Ruslan Yu. Rubtsov[4], Sergey L. Khursan[3], Alexander A. Stepanov[1], Sergey F. Vasilevsky[1], Nikolay E. Polyakov[1,2], Tatyana V. Leshina[1]

[1] Voevodsky Institute of Chemical Kinetics and Combustion, 630090 Novosibirsk, Russia;
[2] Department of Natural Sciences, Novosibirsk State University, 630090 Novosibirsk, Russia;
[3] Ufa Institute of Chemistry, Ufa Federal Research Centre of the Russian Academy of Sciences, 450054 Ufa, Russia;
[4] The Institute of Problems of Chemical Physics of the Russian Academy of Sciences, 142432 Chernogolovka, Russia.

*corresponding author: doktorov@kinetics.nsc.ru

1. **Photoinduced processes in dyads.**

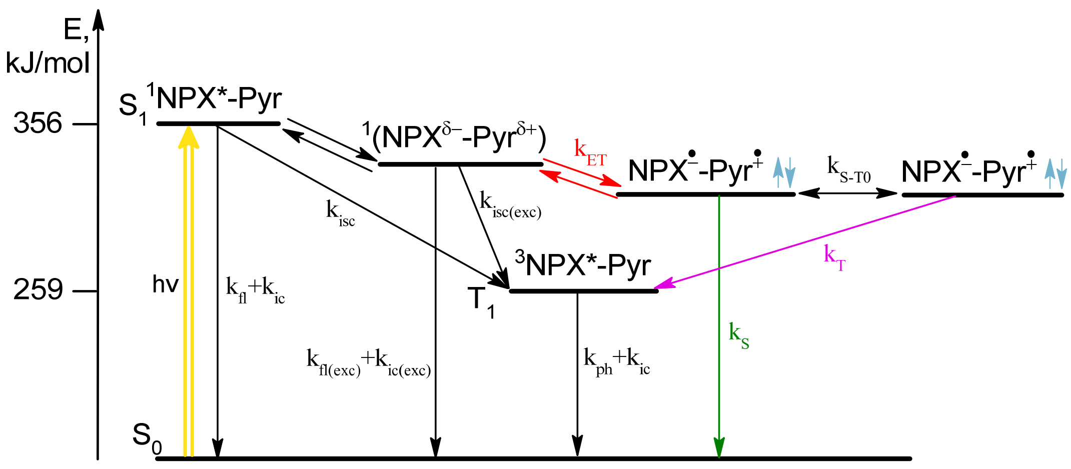

Scheme S1. Photoinduced processes in systems where NPX enantiomers are linked with (S)-N-methylpyrrolidin. Upon photoexcitation, the singlet local excited state (LE) is formed, and then LE converts to an exciplex (partial charge transfer), in which electron transfer (ET) can occur, followed by the formation of a biradical-zwitterion (BZ). The back electron transfer can occur from triplet (k_T) and singlet (k_S) collective spin states of BZ. From experimental data k_S was shown to be higher than k_T. Subscripts denote fl – fluorescence, ic – internal conversion, isc – intersystem crossing, ph – phosphorescence, exc – exciplex, S-T_0 – intersystem crossing in BZ.

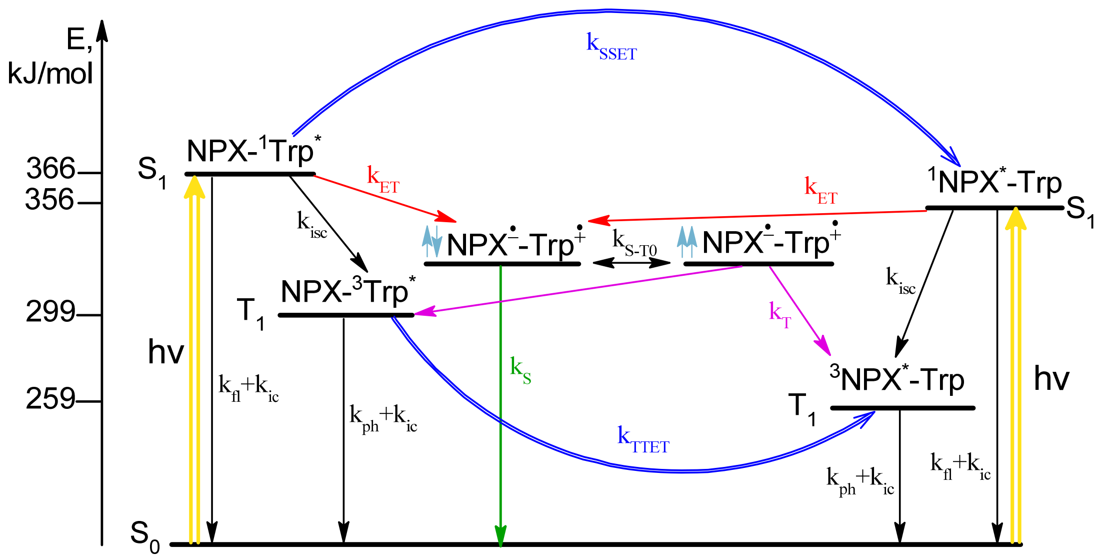

Scheme S2. Photoinduced processes in NPX-Trp dyad. The fraction of light absorbed by aminoacid and drug molecules depends on excitation wavelength, in CIDNP experiments λ_{exc}=308 nm and all light is absorbed by NPX (97%), at λ_{exc}=300 nm in fluorescence experiments 40% of light is absorbed by Trp and 60% by NPX. Upon photoexcitation the singlet excited state of chromophore is formed, then electron transfer may occur both from Trp in ground state to ^{1}NPX* in singlet excited state and from singlet excited state of ^{1}Trp* to NPX in ground state resulted in formation of BZ. From experimental data, the rate of back electron transfer from triplet collective spin state (k_T) of BZ was established to be higher than from singlet (k_S). In addition, the singlet ^{1}Trp* and triplet ^{3}Trp*excited states are quenched *via* singlet-singlet (SSET) and triplet-triplet energy transfer (TTET), correspondingly. In essence, photoinduced processes related with CIDNP is limited by the right part of scheme that describes ^{1}NPX* quenching.

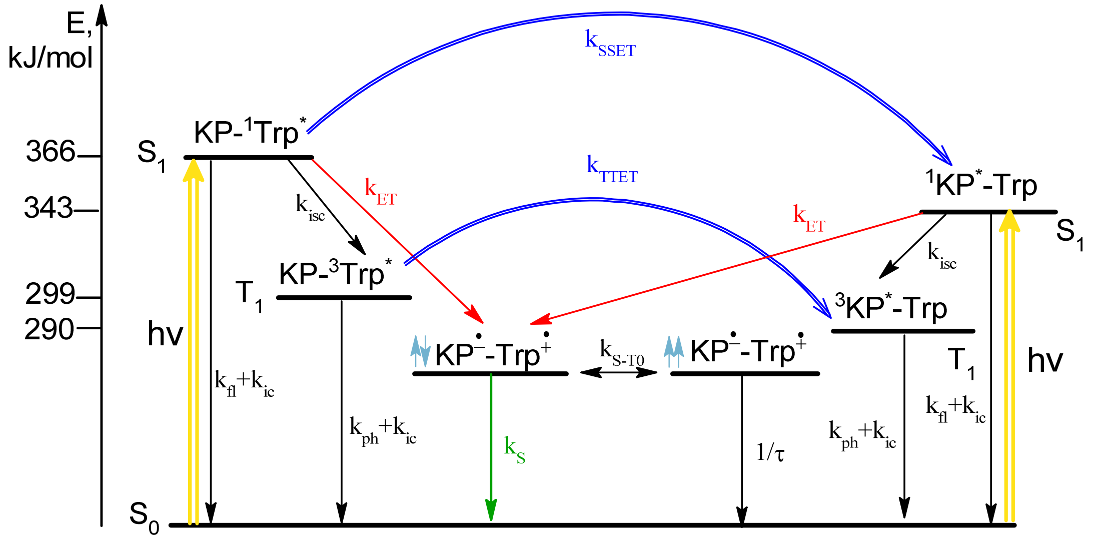

Scheme S3. Photoinduced processes in the KP-Trp dyad. The fraction of light absorbed at λ_{exc}=308 nm is 55/45 KP/Trp, at λ_{exc}=280 nm 65/35 KP/Trp. This scheme is similar to the NPX-Trp case,

the difference is that back ET is allowed only from the singlet spin state of BZ. Experimental data showed that CIDNP effects are formed as a result of ET from excited singlet state of $^{1}Trp^{*}$ to KP in ground state. As appears from above, when discussing the CIDNP effects, only the left part of photoinduced processes should be considered. Energy of singlet and triplet excited states of KP are taken from Lhiaubet, V., Gutierrez, F., Penaud–Berruyer, F., Amouyal, E., Daudey, J.-P., Poteau, R., … Paillous, N. (2000). Spectroscopic and theoretical studies of the excited states of fenofibric acid and ketoprofen in relation with their photosensitizing properties. New Journal of Chemistry, 24(6), 403–410. Musa, K. A. K., Matxain, J. M., & Eriksson, L. A. (2007). Mechanism of Photoinduced Decomposition of Ketoprofen. Journal of Medicinal Chemistry, 50(8), 1735–1743.

2. Calculation of CIDNP in the frame of S –T_0 approximation.

Table S1. Calculated values of CIDNP effects on protons of dyad I and II. Calculation parameters $\Delta g = 6x10^{-4}$; magnetic field 47000 G; diffusion coefficient D = 10^{-6} cm^2/s; Onsager radius 15 A, contact radius 7 A, τ – lifetime of BZ. Values of corresponding HFI constants are following:

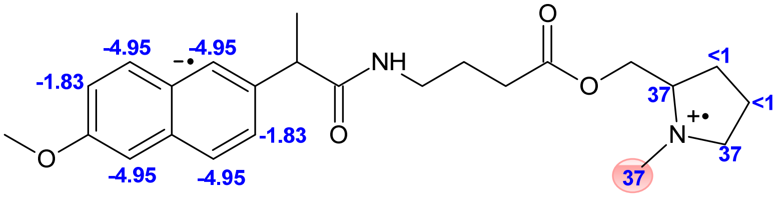

τ (ns)	CIDNP of aromatic protons	CIDNP of N-CH_3
	4 nuclei (a = -4.95G)	3 nuclei (a_{eff} = 37G)
7	-0.0051	-0.0067
9	-0.0048	-0.0057
14	-0.0039	-0.0039
20	-0.0032	-0.0030
35	-0.0020	-0.0016
70	-0.0012	-0.0009

Table S2. Calculated values of CIDNP effects on protons of dyad III. Calculation parameters Δg = 4x10^{-4}; magnetic field 47000 G; diffusion coefficient D = 10^{-6} cm2/s; Onsager radius 15 A, contact radius 7 A, τ – lifetime of BZ. Values of corresponding HFI constants are following:

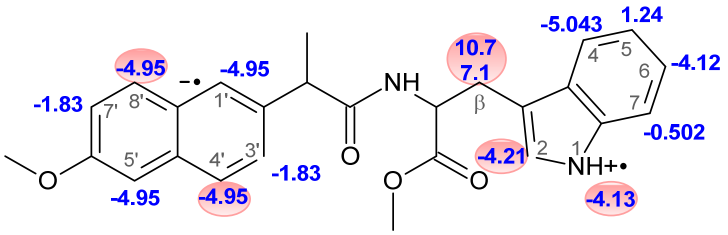

τ (ns)	Aromatic protons of NPX	Protons of Trp moiety				
	1’, 4’, 8’, 5’ (a=-4.95G) 4 nuclei	β-CH_2 average for 2 nuclei (a_1=10.7G a_2=7.1G)	2-CH (a=-4.2 G)	1-NH (a=-4.1 G)	4-CH (a=-5.0 G)	6-CH (a=-4.1 G)
7	-0.0201	-0.0100	0.0041	0.0039	0.0050	0.0039
10	-0.0188	-0.0094	0.0038	0.0036	0.0047	0.0036
14	-0.0167	-0.0084	0.0033	0.0031	0.0041	0.0032
20	-0.0135	-0.0068	0.0027	0.0025	0.0034	0.0025
35	-0.0096	-0.0049	0.0019	0.0017	0.0024	0.0017
50	-0.0070	-0.0036	0.0014	0.0013	0.0018	0.0013
100	-0.0038	-0.0019	0.0007	0.0007	0.0009	0.0007

Table S3. Calculated values of CIDNP effects on protons of dyad IV. Calculation parameters Δg = 6x10^{-4}; magnetic field 47000 G; diffusion coefficient D = 10^{-6} cm^2/s; Onsager radius 15 A, contact radius 7 A, τ – lifetime of BZ. Values of corresponding HFI constants are following:

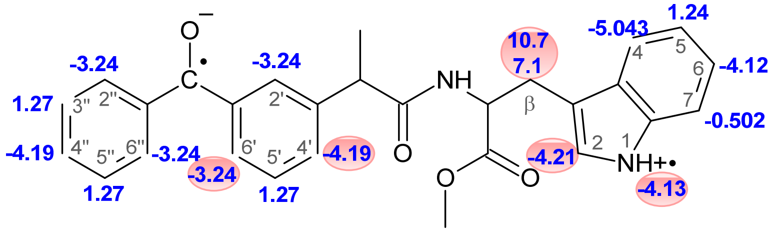

τ (ns)	Aromatic protons of KP		Protons of Trp moiety				
	2’, 6’, 2’’, 6’’ (a=-3.24) 4 nuclei	4', 4" (a=-4.19) 2 nuclei	β-CH_2 average for 2 nuclei (a_1=10.7G a_2=7.1G)	2CH (a=-4.2)	1NH (a=-4.1)	4CH (a=-5.0)	6CH (a=-4.1)
7	0.0074	0.0050	0.0055	-0.0025	-0.0024	-0.0031	-0.0024
10	0.0073	0.0050	0.0054	-0.0025	-0.0024	-0.0031	-0.0024
14	0.0068	0.0047	0.0051	-0.0024	-0.0023	-0.0029	-0.0023
20	0.0061	0.0042	0.0046	-0.0021	-0.0020	-0.0026	-0.0020
35	0.0046	0.0032	0.0035	-0.0016	-0.0016	-0.0020	-0.0016
70	0.0031	0.0021	0.0023	-0.0011	-0.0010	-0.0013	-0.0010
100	0.0022	0.0015	0.0017	-0.0008	-0.0007	-0.0010	-0.0007

3. Calculation of CIDNP in dyads within the two-position model.

To elucidate the influence of the magnetic dipole-dipole interaction on the magnitude of the stationary nuclear polarization within the framework of the two-position model, solutions of the equations for the elements of the stationary density matrix are found, which describe quantum transitions between the singlet and triplet spin states under the action of spin interactions. These interactions are: the Zeeman interaction of the electrons of the dyad with an external strong magnetic field, HFI and magnetic dipole-dipole interaction of electrons. A model is considered in which there are two nuclei, each of which interacts with an electron 1 and 2 with HFI constants a_1 and a_2, respectively. The Zeeman interaction of nuclei with an external magnetic field was neglected. Introducing basis functions (1),

$$\begin{gathered} S\alpha_{N1}\alpha_{N2}=1,\ S\alpha_{N1}\beta_{N2}=2,\ S\beta_{N1}\alpha_{N2}=3,\ S\beta_{N1}\beta_{N2}=4 \\ T_0\alpha_{N1}\alpha_{N2}=5,\ T_0\alpha_{N1}\beta_{N2}=6,\ T_0\beta_{N1}\alpha_{N2}=7,\ T_0\beta_{N1}\beta_{N2}=8 \end{gathered} \quad (1)$$

we have equations for the density matrix for transitions between states 1 and 5 (2).

$$\begin{aligned}
-\frac{1}{4} &= -i\left(\mathrm{H}_{15}\rho_{51}^{(1)}-\rho_{15}^{(1)}\mathrm{H}_{51}\right)-k_S\rho_{11}^{(1)}-\frac{1}{\tau_1}\rho_{11}^{(1)}+\frac{1}{\tau_2}\rho_{11}^{(2)} \\
0 &= -i\left(\mathrm{H}_{15}\rho_{51}^{(2)}-\rho_{15}^{(2)}\mathrm{H}_{51}\right)-\frac{1}{\tau_2}\rho_{11}^{(2)}+\frac{1}{\tau_1}\rho_{11}^{(1)} \\
0 &= -i\left(\mathrm{H}_{55}^{(1)}\rho_{51}^{(1)}+\mathrm{H}_{51}\rho_{11}^{(1)}-\rho_{55}^{(1)}\mathrm{H}_{51}\right)-\frac{k_S+k_T}{2}\rho_{51}^{(1)}-\frac{1}{\tau_1}\rho_{51}^{(1)}+\frac{1}{\tau_2}\rho_{51}^{(2)} \\
0 &= -i\left(\mathrm{H}_{55}^{(2)}\rho_{51}^{(2)}+\mathrm{H}_{51}\rho_{11}^{(2)}-\rho_{55}^{(2)}\mathrm{H}_{51}\right)-\frac{1}{\tau_2}\rho_{51}^{(2)}+\frac{1}{\tau_1}\rho_{51}^{(1)} \\
0 &= -i\left(\mathrm{H}_{15}\rho_{55}^{(1)}-\rho_{11}^{(1)}\mathrm{H}_{15}-\rho_{15}^{(1)}\mathrm{H}_{55}^{(1)}\right)-\frac{k_S+k_T}{2}\rho_{15}^{(1)}-\frac{1}{\tau_1}\rho_{15}^{(1)}+\frac{1}{\tau_2}\rho_{15}^{(2)} \\
0 &= -i\left(\mathrm{H}_{15}\rho_{55}^{(2)}-\rho_{11}^{(2)}\mathrm{H}_{15}-\rho_{15}^{(2)}\mathrm{H}_{55}^{(2)}\right)-\frac{1}{\tau_2}\rho_{15}^{(2)}+\frac{1}{\tau_1}\rho_{15}^{(1)} \\
0 &= -i\left(\mathrm{H}_{51}\rho_{15}^{(1)}-\rho_{51}^{(1)}\mathrm{H}_{15}\right)-k_T\rho_{55}^{(1)}-\frac{1}{\tau_1}\rho_{55}^{(1)}+\frac{1}{\tau_2}\rho_{55}^{(2)} \\
0 &= -i\left(\mathrm{H}_{51}\rho_{15}^{(2)}-\rho_{51}^{(2)}\mathrm{H}_{15}\right)-\frac{1}{\tau_2}\rho_{55}^{(2)}+\frac{1}{\tau_1}\rho_{55}^{(1)}
\end{aligned} \quad (2)$$

Here, the superscripts indicate the corresponding position, τ_1 and τ_2 the times the dyad is in a given configuration (a given position within a two-position model). The following values are also introduced:

k_S - the rate of transformation of the biradical in the singlet state,

$k_T = k_{T'} + k_p + \frac{1}{T_1} \equiv k_{T'} + \frac{1}{T}$ $\left(\frac{1}{T} = k_p + \frac{1}{T'}\right)$ - triplet transformation rate,

$k_{T'}$ - triplet transformation rate contributing to the desired polarization,

k_p - the rate of formation of a product from a triplet that does not contribute to the desired polarization,

T' - triplet state relaxation time,

$\frac{1}{T}$ - the total transformation rate of the triplet that does not contribute to the desired polarization.

It is believed that the creation of a dyad and recombination (electron transfer (forward and backward)) is carried out only from the first (contact) position. From the second position, these processes are absent. The values of the diagonal values of the Hamiltonian associated with the magnetic dipole-dipole interaction are different in different positions, and the off-diagonal elements responsible for the internal interactions of the radicals of the dyad are assumed to be the same. It is enough to consider the solution of this system. The solution of other necessary systems is obtained by replacing indices 1 by 2 and 5 by 6, indices 1 by 3 and 5 by 7, indices 1 by 4 and 5 by 8.

The CIDNP on the first nucleus is:

$$P_1 = \frac{\Pi_1}{\omega_1} = k_S\left(\rho_{11}^{(1)} + \rho_{22}^{(1)} - \rho_{33}^{(1)} - \rho_{44}^{(1)}\right) + k_T'\left(\rho_{55}^{(1)} + \rho_{66}^{(1)} - \rho_{77}^{(1)} - \rho_{88}^{(1)}\right), \quad (3)$$

Systems of type (2) include all elements of the singlet and triplet spin states for various nuclear configurations. From these systems, by adding the first, second, seventh and eighth equations, it follows:

$$\begin{aligned} &\frac{1}{4}-\frac{1}{T}\rho_{55}^{(1)} = k_S\rho_{11}^{(1)} + k_T'\rho_{55}^{(1)}, \quad \frac{1}{4}-\frac{1}{T}\rho_{66}^{(1)} = k_S\rho_{22}^{(1)} + k_T'\rho_{66}^{(1)}, \\ &\frac{1}{4}-\frac{1}{T}\rho_{77}^{(1)} = k_S\rho_{33}^{(1)} + k_T'\rho_{77}^{(1)}, \quad \frac{1}{4}-\frac{1}{T}\rho_{88}^{(1)} = k_S\rho_{44}^{(1)} + k_T'\rho_{88}^{(1)}. \end{aligned} \quad (4)$$

Then from (3) and (4) we have, respectively:

$$P_1 = \frac{\Pi_1}{\omega_1} = -\frac{1}{T}\left(\rho_{55}^{(1)} + \rho_{66}^{(1)} - \rho_{77}^{(1)} - \rho_{88}^{(1)}\right), \quad P_2 = \frac{\Pi_2}{\omega_2} = -\frac{1}{T}\left(\rho_{55}^{(1)} + \rho_{77}^{(1)} - \rho_{66}^{(1)} - \rho_{88}^{(1)}\right), \quad (5)$$

That is, polarization is expressed in terms of triplet populations and is proportional to relaxation from these states.

Introducing the notation

$$\frac{\omega_1-\omega_2}{2} = \delta; \qquad \frac{a_1-a_2}{4} = a_-; \qquad \frac{a_1+a_2}{4} = a_+ \quad (6)$$

for the matrix elements of the spin Hamiltonian, we have

$$\begin{aligned} &\mathrm{H}_{55}^{(k)} = \mathrm{H}_{66}^{(k)} = \mathrm{H}_{77}^{(k)} = \mathrm{H}_{88}^{(k)} = -A^{(k)} \quad (k=1,2), \\ &\mathrm{H}_{15} = \mathrm{H}_{51} = \delta + a_- \quad \mathrm{H}_{26} = \mathrm{H}_{62} = \delta + a_+ \quad \mathrm{H}_{37} = \mathrm{H}_{73} = \delta - a_+ \quad \mathrm{H}_{48} = \mathrm{H}_{84} = \delta - a_-. \end{aligned} \quad (7)$$

In our consideration, for simplicity, we will take $a_1 = a_2 = 2a$ and

$$a_- = 0, \quad a_+ = a, \quad \mathrm{H}_{15} = \mathrm{H}_{48} = \delta, \quad \mathrm{H}_{26} = \delta + a, \quad \mathrm{H}_{37} = \delta - a \tag{8}$$

Since all diagonal elements of the spin Hamiltonian in each position are the same, then $\rho_{55}^{(1)} = \rho_{88}^{(1)}$ and from (5) we also have

$$P_1 = -P_2 = -\frac{1}{T}\left(\rho_{66}^{(1)} - \rho_{77}^{(1)}\right). \tag{9}$$

Let us introduce the notation:

$$K = \frac{k_S + k_T}{2}, \quad \Delta = \frac{2\delta a}{\delta^2 + a^2} \qquad \left(a = \frac{a_1}{2} = \frac{a_1}{2}, \quad \delta = \frac{\omega_1 - \omega_2}{2}\right) \tag{10}$$

and dimensionless parameters:

$$Z_0 = \frac{4K\left(\delta^2 + a^2\right)\left[\left(A_1 - A_2\right)^2 \tau_1^{-1} + K\left(K\tau_1^{-1} + \left(\tau_1^{-1} + \tau_2^{-1}\right)^2 + A_2^2\right)\right]}{k_S k_T\left[\left(K\tau_2^{-1} - A_1 A_2\right)^2 + \left(A_2\left(K + \tau_1^{-1}\right) + A_1\tau_2^{-1}\right)^2\right]}$$

$$Z_1 = \frac{4K\left(\delta^2 + a^2\right)}{\left(A_1 - A_2\right)^2 \tau_1^{-1} + K\left(K\tau_1^{-1} + \left(\tau_1^{-1} + \tau_2^{-1}\right)^2 + A_2^2\right)}$$

$$Z_2 = \frac{4\left(\delta^2 + a^2\right)\left(A_1^2 + K\left(K + \tau_1^{-1}\right)\right)}{\left(K\tau_2^{-1} - A_1 A_2\right)^2 + \left(A_2\left(K + \tau_1^{-1}\right) + A_1\tau_2^{-1}\right)^2}$$

(11)

As a result of solving systems of equations of the form (2) from (9), we obtain

$$P_1 = -P_2 = -\frac{Z_0\Delta}{4KT}\left\{\frac{1 + 2Z_1 + Z_1 Z_2\left(1 - \Delta^2\right)}{1 + 2\left(Z_0 + Z_2\right) + 2Z_0 Z_1\left(1 + \Delta^2\right) + \left(1 - \Delta^2\right)\left[\left(Z_2 + Z_0\left(1 + Z_1\right)\right)^2 - Z_0^2 Z_1^2 \Delta^2\right]}\right\}$$

(12)

Note that at, expression (12) is greatly simplified

$$P_1 = -P_2 = -\frac{Z_0\Delta}{4KT\left(\left(1 + Z_0\right)^2 - Z_0^2\Delta^2\right)}$$

(13)

and does not depend on the parameter $Z_1 = Z_2$. That is why, in the absence of motion $\left(\tau_1 = \tau_2 = \infty\right)$, when it follows from (11)

$$Z_0 = Z = \frac{4K^2\left(\delta^2 + a^2\right)}{k_S k_T \left(A_1^2 + K^2\right)}, \quad Z_1 = Z_2 = \frac{4\left(\delta^2 + a^2\right)}{A_2^2},$$

(14)

we have the same result (15):

$$P_1 = -P_2 = -\frac{Z\Delta}{4KT\left(\left(1+Z\right)^2 - Z^2\Delta^2\right)},$$

(15)

which, of course, does not depend on the magnitude of the magnetic dipole-dipole interaction A_2 in the second position.

4. Quantum-chemical calculations

Molecular structure of R-S-isomer KP-Trp in Cartesian coordinates (x, y, z)

Atom	x	y	z
6	-8.396421738	-7.832925603	10.030711657
6	-7.138866093	-8.245199401	9.603940949
6	-6.107866250	-8.293266295	10.533949270
6	-6.314684973	-7.900581151	11.850470673
6	-7.539234304	-7.386283736	12.231032223
6	-8.586748116	-7.357058669	11.314539011
6	-6.876552979	-8.435388929	8.133342934
6	-6.849503347	-7.092325936	7.378056108
8	-6.931060236	-7.053581815	6.163185989
7	-6.687024028	-5.968483560	8.118150608
6	-8.126823114	-4.319036418	6.943614547
6	-9.189344091	-4.266522842	7.986888542
7	-10.940064142	-4.844203416	9.274155895
6	-10.025752205	-5.280792517	8.345204699
6	-9.590954784	-3.126096346	8.760476955
6	-10.685570198	-3.526592283	9.555689652
6	-11.334052913	-2.646042205	10.416299785
6	-10.868337152	-1.350699829	10.473253150
6	-9.782150215	-0.930318999	9.689686482
6	-9.144815294	-1.803801731	8.834474465
6	-6.725232314	-4.671016003	7.491800667
6	-6.291747714	-3.628466539	8.495012436
6	-9.857926439	-6.635186811	11.607772561
8	-10.945097773	-7.090015365	11.317262887
8	-6.197901363	-3.797171383	9.683697365
8	-6.032088652	-2.469328375	7.885634862
6	-5.612689520	-1.411385878	8.749992568
6	-9.685466120	-5.275241889	12.187063387
6	-8.602411535	-4.491725772	11.794395572
6	-8.456796400	-3.208906478	12.286473222
6	-9.375359310	-2.714411921	13.198812680
6	-10.459369248	-3.488792669	13.590382517
6	-10.628074565	-4.761039247	13.069923165
6	-7.855256330	-9.381093367	7.441548383
1	-9.225837389	-7.794156640	9.342390985
1	-5.121898768	-8.609151059	10.219712428

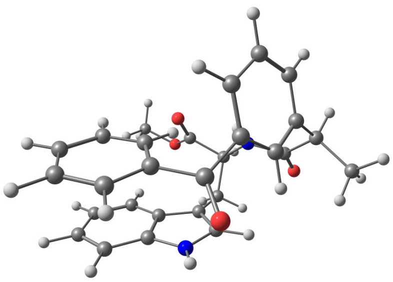

1	-5.500959673	-7.942952649	12.559861957
1	-7.680722380	-6.990861679	13.226623523
1	-5.868960556	-8.838667680	8.009791889
1	-6.682647542	-5.999212718	9.124655722
1	-8.358186765	-5.075153718	6.197426334
1	-8.052171393	-3.360880946	6.429648876
1	-11.550388142	-5.447938275	9.802130146
1	-10.079270198	-6.280328591	7.950797633
1	-12.175584162	-2.964214888	11.013666744
1	-11.355489787	-0.642514420	11.127874471
1	-9.457008776	0.099016663	9.743482070
1	-8.337466970	-1.465854415	8.200365447
1	-6.028119485	-4.652758071	6.654823704
1	-6.388949954	-1.197317070	9.481158586
1	-5.440896527	-0.556084305	8.106149610
1	-4.698941791	-1.693730706	9.266559284
1	-7.885395236	-4.858171834	11.076319159
1	-7.631985656	-2.605298647	11.944977681
1	-9.259871730	-1.713778725	13.590824065
1	-11.177235292	-3.094989338	14.295513800
1	-11.475775653	-5.370277877	13.350037833
1	-8.877353288	-9.013423032	7.512340477
1	-7.603681696	-9.461776798	6.388299657
1	-7.812954984	-10.365326719	7.903655774

Molecular structure of S-R-isomer KP-Trp in Cartesian coordinates (x, y, z)

Atom	x	y	z
6	17.036736983	7.254249675	8.702071484
6	16.142455923	7.675447786	7.723986121
6	16.653893740	8.162303232	6.527833674
6	18.022646357	8.189950577	6.294150254
6	18.891640994	7.662047439	7.229198061
6	18.394117217	7.196034954	8.443661726
6	14.669191256	7.417818919	7.897244896
6	14.325267142	5.922341691	7.767965744
8	13.241717420	5.496907574	8.127963479
7	15.258971821	5.116507374	7.207283808
6	15.069677292	3.004101658	8.499884797
6	16.369171274	3.143275310	9.214982140
7	17.943362198	3.870975746	10.646826411
6	16.692582391	4.103378317	10.126629682
6	17.501475700	2.266061102	9.140659103
6	18.468121052	2.755873826	10.044320898
6	19.692321846	2.120983502	10.230532631
6	19.938218433	0.980309041	9.498316975
6	18.988268412	0.472567141	8.598779222
6	17.775614453	1.102434978	8.416884763
6	15.036936433	3.695424454	7.118139539
6	16.083364225	3.090137667	6.214198810
6	19.253203037	6.434818000	9.397516296
8	19.210950653	6.614764812	10.597589294
8	17.089495440	3.640520916	5.845887476
8	15.751325077	1.841563092	5.879531647
6	16.683665555	1.172200216	5.028610074
6	20.082531792	5.365668541	8.774676273
6	19.578473807	4.652021292	7.688830144
6	20.306938731	3.616215888	7.136935650
6	21.559588315	3.311501712	7.643464361
6	22.067156480	4.017270963	8.725730895
6	21.322239898	5.029374114	9.307577803
6	14.090628611	7.947204723	9.207517182
1	16.679958538	6.879919047	9.648658281
1	15.974699999	8.493224050	5.753845559

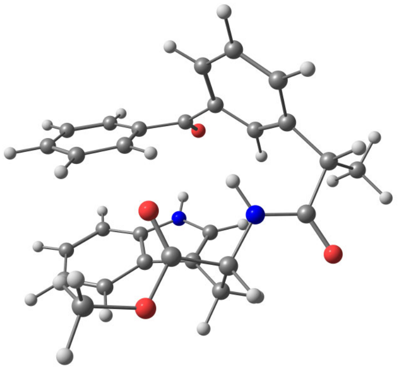

1	18.401616909	8.569504749	5.356631301
1	19.948743577	7.591706060	7.015238144
1	14.134579722	7.893148619	7.072207049
1	16.167203267	5.464412680	6.944322651
1	14.828495431	1.951826167	8.350896835
1	14.263621693	3.447374436	9.079696743
1	18.461757489	4.532187889	11.204081510
1	16.086867979	4.912221243	10.496287033
1	20.421032661	2.505310813	10.928933874
1	20.877739667	0.463006468	9.625901893
1	19.205222756	-0.437030404	8.057463588
1	17.032551115	0.688145679	7.750403755
1	14.056512741	3.510672072	6.680899208
1	16.787760728	1.713712842	4.092081424
1	17.652965901	1.104519036	5.516874464
1	16.268427222	0.185624533	4.856545902
1	18.601019846	4.872319412	7.288534775
1	19.884963767	3.056418220	6.319629484
1	22.136569026	2.509045981	7.207437167
1	23.041677949	3.770733687	9.121494338
1	21.694805659	5.574946301	10.163161108
1	14.576537122	7.489274605	10.067198699
1	14.230644953	9.024271727	9.270075909
1	13.031799692	7.713072511	9.259641966

Molecular structure of S-S-isomer KP-Trp in cartesian coordinates (x, y, z)

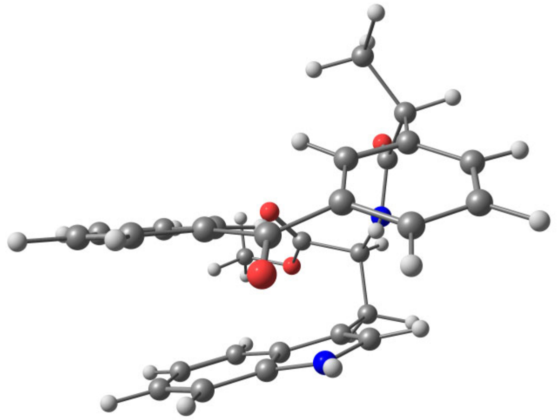

Atom	x	y	z
6	-11.164795021	-13.934432543	9.438498124
6	-10.003829522	-14.599284590	9.826917109
6	-10.049800211	-15.388795221	10.968765667
6	-11.222616884	-15.507854666	11.706715115
6	-12.378930713	-14.887700111	11.277174335
6	-12.362707509	-14.117006027	10.116344902
6	-8.772276045	-14.549471408	8.953028578
6	-8.868766210	-15.450844277	7.709211143
8	-7.984390650	-15.473341046	6.877361854
7	-9.968245613	-16.244983903	7.619554824
6	-11.383769484	-17.983499311	6.683191629
6	-12.608241473	-17.346430397	7.257616177
7	-14.094839018	-16.538587465	8.734938454
6	-12.893819770	-17.191219154	8.584389122
6	-13.689798849	-16.732929231	6.538334982
6	-14.594537028	-16.231654704	7.495227609
6	-15.734706798	-15.513684607	7.138409803
6	-15.960177087	-15.313563866	5.795746194
6	-15.083053911	-15.824424137	4.822940511
6	-13.957179479	-16.531756828	5.180281298
6	-10.247708904	-16.978976819	6.419529321
6	-10.593230822	-16.056830719	5.258693972
6	-13.678356043	-13.594544778	9.624371998
8	-14.648359095	-13.656562971	10.357015458
8	-10.789799298	-14.874655578	5.322898389
8	-10.647689415	-16.759549420	4.119880792
6	-10.913285410	-15.991562660	2.946863207
6	-13.836579068	-13.063981331	8.240353684
6	-13.104567733	-13.520188214	7.145525140
6	-13.396732517	-13.068488955	5.869906275
6	-14.431684165	-12.170036475	5.674733113
6	-15.183879256	-11.730098849	6.755719620
6	-14.888949045	-12.175381513	8.029658083
6	-8.379293768	-13.141893205	8.504329313
1	-11.128059250	-13.282442957	8.582647065
1	-9.162186295	-15.918990908	11.287375933

1	-11.232413406	-16.105082921	12.607238520	
1	-13.306849511	-14.987335563	11.820245907	
1	-7.936730855	-14.967586681	9.517291602	
1	-10.759525021	-16.038208388	8.206792710	
1	-11.000936784	-18.732920733	7.374128466	
1	-11.618131445	-18.488365459	5.748137393	
1	-14.473996779	-16.207256257	9.606181209	
1	-12.333561514	-17.512200720	9.447415759	
1	-16.395311680	-15.104998487	7.889168929	
1	-16.824892022	-14.746415718	5.484339963	
1	-15.299826813	-15.657577563	3.777755209	
1	-13.287835191	-16.926519326	4.428222835	
1	-9.351440032	-17.515601204	6.113843077	
1	-10.152988531	-15.224843888	2.823955242	
1	-10.889856233	-16.692641039	2.120504227	
1	-11.889168597	-15.516541321	3.022654564	
1	-12.334469012	-14.266584213	7.250832491	
1	-12.817032698	-13.444153210	5.040977024	
1	-14.659776473	-11.816944878	4.679173277	
1	-15.995904700	-11.033907970	6.603035138	
1	-15.472288310	-11.853291441	8.878495361	
1	-9.149030664	-12.696858755	7.876697770	
1	-8.221461305	-12.501310805	9.369742354	
1	-7.465337438	-13.193776788	7.920110077	
